# Sleep deprivation-induced sympathetic activation promotes pro-tumoral macrophage phenotype via the ADRB2/KLF4 pathway to facilitate NSCLC metastasis

**DOI:** 10.1016/j.isci.2025.112321

**Published:** 2025-03-30

**Authors:** Shuxian Yin, Jiali Wang, Yunlong Jia, Xiaoyi Wang, Yan Zhao, Tianxu Liu, Wei Lv, Yuqing Duan, Song Zhao, Sheng Wang, Lihua Liu

**Affiliations:** 1 Department of Tumor Immunotherapy, Fourth Hospital of Hebei Medical University and Hebei Cancer Research Institute, Shijiazhuang, China; 2 Hebei Key Laboratory of Stomatology, Hebei Technology Innovation Center of Oral Health, Hebei Medical University, Shijiazhuang, China; 3 Hebei Key Laboratory of Neurophysiology, Hebei Medical University, Shijiazhuang, China; 4 Department of Pathology, Hebei Medical University, Shijiazhuang, China; 5 International Cooperation Laboratory of Stem Cell Research, Hebei Medical University, Shijiazhuang, China

**Keywords:** Microenvironment, Natural sciences, Biological sciences, Physiology, Systems biology, Cancer systems biology

## Abstract

Sleep deprivation is one of concomitant symptoms of cancer patients, particularly those with non-small cell lung cancer (NSCLC). The potential effect of sleep deprivation on tumor progression and underlying mechanisms remain to be fully investigated. Using a sleep-deprived tumor-bearing mouse model, we found that sleep deprivation altered immune cell composition and regulated pro-tumoral M2 macrophage polarization by the sympathetic nervous system. Furthermore, we identified a role of catecholaminergic neurons in the rostral ventrolateral medulla (RVLM) in influencing NSCLC metastasis. Clinical analyses revealed a correlation between sympathetic-related indicators and poor prognosis. Mechanistically, our findings indicate that sleep deprivation facilitates the polarization of pro-tumoral macrophages by upregulating β2-adrenergic receptor (ADRB2), which subsequently enhances the expression of Kruppel-like transcription factor 4 (KLF4) through the JAK1/STAT6 phosphorylation pathway. These findings highlight a neuro-immune mechanism linking sleep deprivation to NSCLC metastasis, suggesting that targeting the ADRB2/KLF4 axis could improve outcomes for sleep-deprived NSCLC patients.

## Highlights

• Sleep deprivation drives M2 macrophage polarization via sympathetic activation
• Catecholaminergic neurons in the RVLM regulate sleep deprivation-induced NSCLC metastasis
• Sympathetic activation regulates ADRB2-JAK1/STAT6 phosphorylation-KLF4 in macrophages
• Sympathetic indicators linked to poor prognosis in sleep-deprived NSCLC patients

## Graphical abstract

**Figure:**
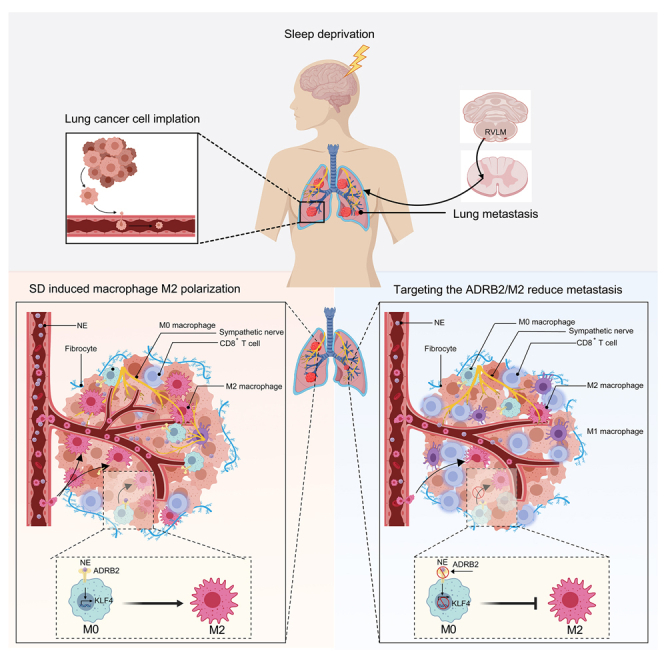

## Introduction

Insomnia is a common symptom in modern society, with an estimated prevalence ranging from 10% to 40%.^1^ The high levels of anxiety caused by survival pressure often lead cancer patients to suffer from insomnia as one of the most common accompanying symptoms.^2^ Sever insomnia can result in sleep deprivation which refers to an insufficient effective sleep time-caused inadequacy for maintaining psychological and physiological homeostasis.^3^^,^^4^^,^^5^ For cancer patients, sleep deprivation is not only a problem that affects the quality of life, but also correlated with occurrence and mortality.^6^ In lung cancer, sleep deprivation is correlated with a 13% higher risk of occurrence and an elevated risk of mortality.^7^^,^^8^^,^^9^^,^^10^ As for non-small cell lung cancer (NSCLC), the dominant pathological subtype of lung cancer, reduced sleep was reported to be correlated with a 21% increase in cancer-specific mortality.^11^ Thus, sleep deprivation is an important clinical symptom that plagues NSCLC patients and is correlated with unsatisfactory outcomes.^12^ However, the specific mechanisms involved in sleep deprivation-mediated progression of NSCLC remain to be elucidated, which restricts the establishment of novel therapeutic strategies.

Sleep deprivation induces physiological stress, resulting in decreased parasympathetic tone and increased sympathetic tone.^13^ Research indicates that during wakefulness, adrenergic neurons in the rostral ventrolateral medulla (RVLM) are activated, resulting in increased sympathetic nerve excitability.^14^ Thus, during the stress response, specific neural circuits are activated, which in turn stimulate the peripheral sympathetic nervous system via the sympathetic premotor regions in the ventral brainstem.^15^ Neuro-immunological studies have demonstrated that the sympathetic nervous system functions as a conduit for centrally regulating immune function, thus, sleep deprivation impacts immune cell activity and intercellular communication through altering the landscape of immune cells.^16^ Activation of catecholaminergic neurons in the ventrolateral medulla (VLM) has been implicated in tumor growth regulation via modulation of CD8^+^ T cells.^17^ Previous studies have shown that during sleep deprivation, expression of GABA_A_ receptor in the paraventricular nucleus of the hypothalamus (PVH) decreases, while excitability of corticotropin-releasing hormone (CRH) neurons within the PVH increases. PVH regulates the migration of monocytes and lymphocytes from secondary lymphoid organs and blood to bone marrow, suppressing adaptive immunity.^18^^,^^19^ Consequently, sleep deprivation reshapes the tumor microenvironment, decreasing anti-tumor CD3^+^ T cells and natural killer (NK) cells while increasing immunosuppressive cells like Tregs and M2 macrophages.^20^^,^^21^ Additionally, sleep deprivation reduces serum interferon gamma (IFN-γ)/interleukin-4 (IL-4) and IFN/IL-10 ratios, potentially promoting tumorigenesis and metastasis.^22^^,^^23^^,^^24^ These findings underscore sleep deprivation as a stressor that accelerates tumor progression by modulating tumor immunity via the nervous system’s influence on immune regulation. However, the molecular mechanisms through which sleep deprivation remodels the immune microenvironment remain elusive.

In this study, we identified that sleep deprivation exacerbated the growth and metastasis of NSCLC. This effect was attributed to its capacity to increase the excitability of peripheral sympathetic nervous system and activate catecholaminergic neurons in the RVLM followed by an elevated proportion of the pro-tumoral macrophages in tumor microenvironment. Mechanistically, activated peripheral sympathetic nervous system upregulated the genes associated with the pro-tumoral phenotype in macrophages through ADRB2 and Kruppel-like transcription factor 4 (KLF4) signaling. These findings suggested that sleep deprivation-stimulated sympathetic nervous system promoted NSCLC progression by remodeling an immunosuppressive tumor microenvironment dominated by the pro-tumoral macrophages.

## Results

### Sleep deprivation promotes the progression of NSCLC

To investigate the effect of sleep deprivation on NSCLC, we established a sleep deprivation model using C57BL/6J mice (Figure S1A). Electroencephalogram (EEG) and electromyography (EMG) data revealed that sleep deprivation increased awake time and reduced total sleep time, including both non-rapid eye movement (NREM) and rapid eye movement (REM) sleep (Figures S1B–S1D). Following 24 h of sleep deprivation, EEG and EMG analysis indicated an increase in both NREM and REM sleep duration, as well as a decrease in awake time in the sleep deprivation group of tumor-bearing mice, suggesting that sleep deprivation affected the overall sleep architecture over the subsequent 24 h (Figures S1E–S1H). The aforementioned results demonstrated that the sleep deprivation model was successfully established.

**Figure 1:**
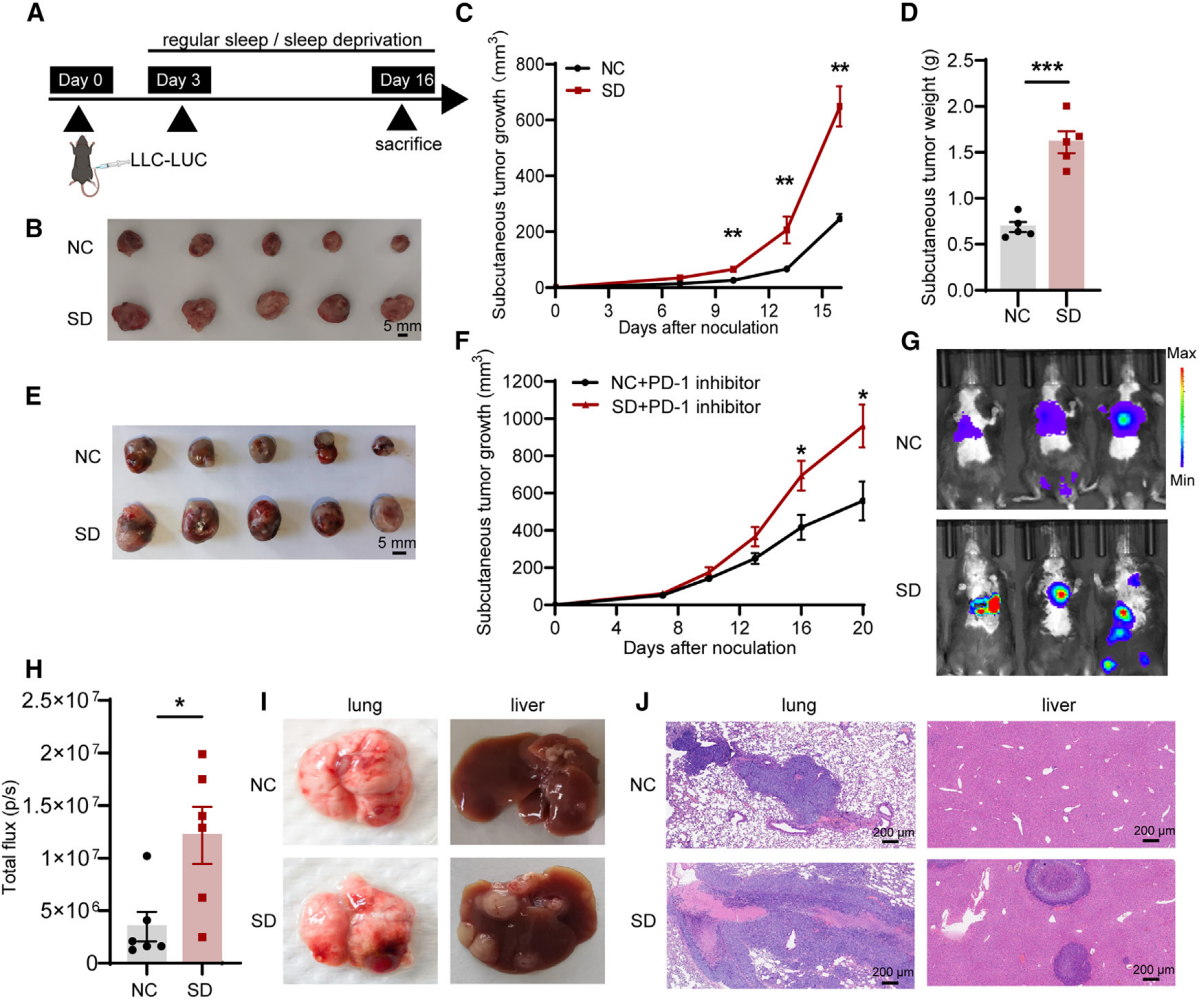
Sleep deprivation promotes the growth and metastasis of NSCLC *in vivo* (A) Schematic diagram of NSCLC subcutaneous xenograft model with sleep deprivation. (B) Subcutaneous tumor comparison in each group. (C and D) Growth and weight of experimental models with subcutaneous xenografts in the NC group and sleep deprivation group (*n* = 5). (E and F) Tumor progression under PD-1 treatment in different sleep conditions (*n* = 5). (G and H) Internal bioluminescence of metastatic foci in the experimental models (*n* = 6). (I and J) Comparison of lung and liver metastatic foci in the experimental groups. Data represent the mean ± SEM. Statistical significance was determined through the Student’s t test or Mann-Whitney U test. ^∗^*p* < 0.05, ^∗∗^*p* < 0.01, and ^∗∗∗^*p* < 0.001.

Next, we established a xenograft tumor mouse model by subcutaneously injecting LLC-LUC cells in the right groin. The findings revealed a significant increase in tumor volume and weight among mice subjected to sleep deprivation, as compared to those in the control group with normal sleep patterns (Figures 1A–1D). Moreover, in mice treated with PD-1 inhibitors, sleep deprivation also promoted tumor growth (Figures 1E and 1F). Furthermore, the sleep-deprived group showed a higher occurrence of pulmonary and liver nodules in the metastasis model compared to the control group with regular sleep (Figures 1G–1J). The aforementioned results demonstrated that sleep deprivation had promoting effect on the progression of NSCLC *in vivo*.

### Sleep deprivation promotes the progression of NSCLC by activating peripheral sympathetic nerve system

**Figure 2:**
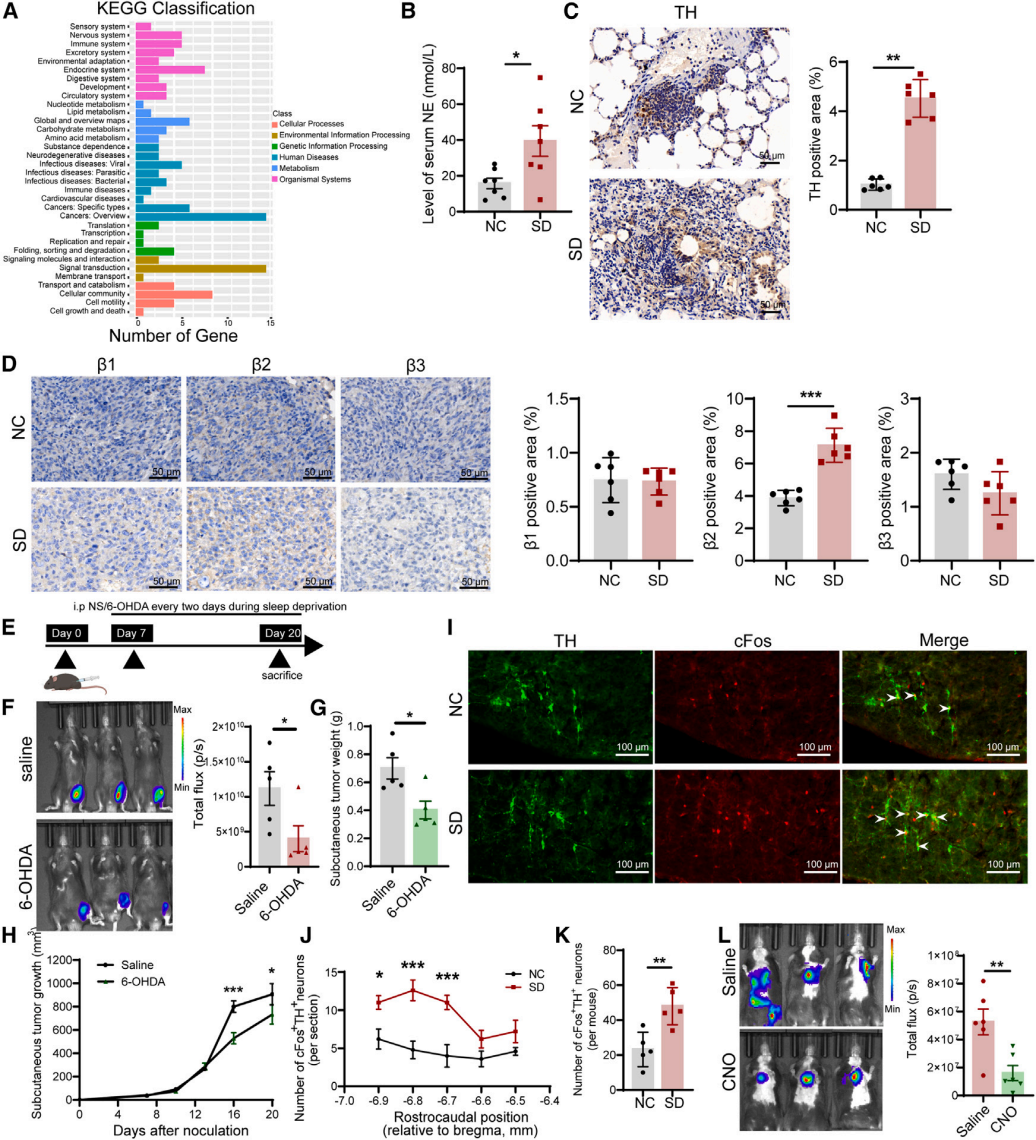
Sleep deprivation promotes NSCLC progression by stimulating peripheral sympathetic system and activating catecholaminergic neurons in RVLM (A) KEGG enrichment analysis of pulmonary metastasis in the NC group and sleep deprivation group (*n* = 3). (B) NE level in serum of NC group and sleep deprivation group (*n* = 7). (C and D) Quantitative expression of TH, β1, β2, and β3 adrenergic receptors in tumor tissue of the NC group and sleep deprivation group. (E–H) Representative live imaging, weight and growth curves of subcutaneous tumors in the saline- and 6-OHDA-treated tumor-bearing mice subject to sleep deprivation (*n* = 5). (I) Representative microscopic images of TH^+^ (green) and cFos^+^ (red) neurons in the RVLM. (J) The number of cFos^+^TH^+^ neurons in the rostrocaudal position (five coronal brain sections per mouse, *n* = 5). (K) The total number of cFos^+^TH^+^ neurons of RVLM in the NC group and sleep deprivation group, five coronal brain sections per mouse (*n* = 5). (L) Bioluminescence quantitative analysis of metastatic tumors in mice transfected with chemogenetics virus *in vivo* (*n* = 6). Data represented as mean ± SEM. Statistical significance was determined through the Student’s t test or Mann-Whitney U test. ^∗^*p* < 0.05, ^∗∗^*p* < 0.01, and ^∗∗∗^*p* < 0.001.

Transcriptome sequencing was employed to elucidate the mechanisms linking sleep deprivation and NSCLC in tumor-bearing mice from both the normal sleep and sleep deprivation group. Analysis of Kyoto encyclopedia of genes and genomes (KEGG) pathways indicated significant enrichment related to the nervous and immune systems, suggesting that sleep deprivation disrupted normal neuro-immune regulation (Figure 2A). Consequently, the level of norepinephrine (NE) in the serum of sleep-deprived mice was evaluated, revealing a significant increase in NE among sleep-deprived tumor-bearing mice compared to those with regular sleep patterns (Figure 2B). With prolonged sleep deprivation, NE expression increased (Figure S2A). Studies have suggested that sleep deprivation was correlated with heightened activity of the sympathetic nervous system. To validate the activation of sympathetic nervous system in the tumor tissue, we observed a significant upregulation of sympathetic nerve marker tyrosine hydroxylase (TH) expression in the tumor tissue of sleep-deprived mice (Figure 2C). Previous research has demonstrated that adrenergic receptors consist of alpha receptors and beta receptors, which are further categorized into two subtypes for alpha receptors (α1 and α2) and three subtypes for beta receptors (β1, β2, and β3). Our findings showed that the expression level of ADRB2 and TH in tumor tissues of sleep-deprived mice was significantly higher than those in the normal sleep group, while the expression levels of ADRA1, ADRA2, ADRB1, and ADRB3 showed no significant changes (Figures 2D and S2B).

Subsequently, we conducted further investigation into the behavioral change of tumor-bearing mice using behavioral testing. Through the open field experiment and the elevated plus maze experiment, the behavioral data showed that sleep deprivation increased the anxiety-like behavior of tumor-bearing mice (Figures S2C–S2F). The Barnes maze was utilized to assess spatial learning and memory in two groups of mice, with no statistically significant differences observed between the groups (Figures S2G and S2H). In the three-chamber social experiment, sleep deprivation did not impact sociability and social novelty, but it reduced social memory (Figures S2I–S2K). Moreover, the CODA non-invasive blood pressure system was utilized for blood pressure measurement, and the findings demonstrated that sleep deprivation led to an increase in both systolic and diastolic blood pressure, as well as average blood pressure (Figure S2L). These results further suggested that sleep deprivation resulted in an increased excitability of the sympathetic nervous system.

To verify the effect of sleep deprivation induced sympathetic nervous system excitability on tumor burden, mice with tumors were divided into two groups during sleep deprivation. One group was given saline via the peritoneal cavity as a control, and the other group was given 6-OHDA for peripheral sympathetic blockade. The findings revealed that the blockage of peripheral sympathetic nerves led to the suppression of tumor growth.(Figures 2E–2H). In conclusion, our findings indicated that activation of the sympathetic nervous system played a pivotal role in promoting the progression of NSCLC during periods of sleep deprivation.

### Sleep deprivation promotes NSCLC metastasis by activating catecholaminergic neurons in the RVLM

The RVLM is a pivotal central region housing catecholaminergic neurons that function as presympathetic neurons, responsible for regulating sympathetic outflow.^25^ To investigate the potential activation of catecholaminergic neurons in the RVLM of tumor-bearing mice due to sleep deprivation, we conducted an immunofluorescence assay to assess the expression of TH and the neuron activation marker cFos in the RVLM region. The results revealed a significant activation of TH-immunoreactive neurons in response to sleep deprivation in tumor-bearing mice (Figure 2I). Further detailed analysis showed that bilateral cFos^+^TH^+^ neurons of the RVLM in sleep deprivation group and control group. These activated neurons were significantly more abundant in the sleep deprivation group (Figures 2J and 2K). These findings suggested that catecholaminergic neurons in RVLM may be involved in increasing sympathetic nervous system excitation induced by sleep deprivation in NSCLC.

Catecholaminergic neurons express solely or doubly TH, dopamine β-hydroxylase (DβH), and phenylethanolamine-N-methyltransferase (PNMT), with PNMT being most widely distributed in the RVLM. To investigate whether catecholaminergic neurons in RVLM participate in sleep deprivation-induced NSCLC metastasis, we used Cre/Loxp strategy to manipulate PNMT^RVLM^ neurons. First, we bilaterally injected AAV-hSyn-DIO-hM4Di-EGFP virus into the RVLM of PNMT-Cre mice (Figure S2M), resulting in the specific expression of hM4Di-EGFP in PNMT^RVLM^ neurons. After two weeks of virus injection, LLC-LUC cells were administered via the tail vein, followed by induction of sleep deprivation one week later. During this period, clozapine N-oxide (CNO) was administered daily via intraperitoneal injection (Figure S2N) for chemogenetic stimulation of PNMT^RVLM^ neurons neurons, and saline was employed as the control group. Immunohistochemical assessment of EGFP expression confirmed the successful transfection of this virus in the catecholaminergic neurons of RVLM region (Figure S2O). Chemogenetics experiment results showed the CNO compound administration mitigated the promotion of NSCLC metastasis induced by sleep deprivation (Figure 2L). These findings suggested that the accelerating effect of sleep deprivation on metastasis of NSCLC was contributed to the excitation of the catecholaminergic neurons in RVLM—a key control center for the sympathetic nervous system.

### Sleep deprivation impairs antitumor immunity within the tumor microenvironment

**Figure 3:**
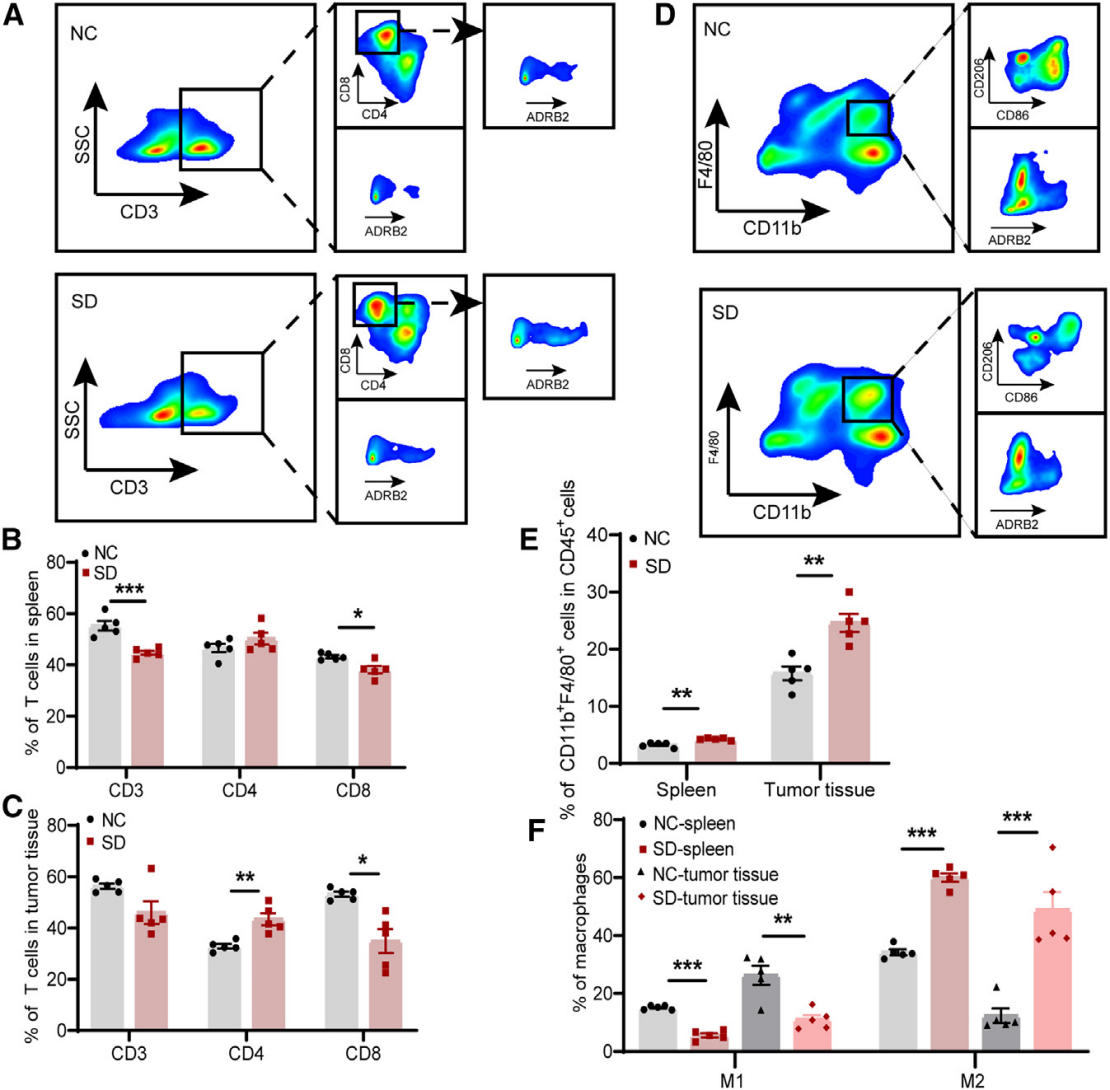
Sleep deprivation suppresses antitumor immunity in tumor microenvironment (A) Flow cytometry plots showing CD3^+^ T, CD4^+^ T, CD8^+^ T cells, and ADRB2 expression of T cells in NC and sleep deprivation groups. (B and C) Statistical analysis of the percentages of CD3^+^ T cells, CD4^+^ T cells, and CD8^+^ T cells in spleen and tumor tissue of NC and sleep deprivation groups. (D) Representative gating strategy in multicolor flow cytometry showing M0 macrophages (CD11b^+^F4/80^+^), M1-polarized macrophages (CD11b^+^F4/80^+^CD86^+^), M2-polarized macrophages (CD11b^+^F4/80^+^CD206^+^) and ADRB2 expression of macrophages in NC and sleep deprivation groups. (E) Proportions of M0 macrophages in spleens and tumor tissues of mice. (F) Quantitative analysis of flow cytometry results showing the percentages of M1-polarized and M2-polarized macrophages within spleens and tumor tissues in each group. Data represented as mean ± SEM. Statistical significance was determined by unpaired, two-tailed Student’s t test or Mann-Whitney U test. ^∗^*p* < 0.05, ^∗∗^*p* < 0.01, and ^∗∗∗^*p* < 0.001.

The relationship between sleep and immunity is bidirectional, and sleep deprivation acts as a stressor that impairs the immune system.^22^ Thus, we performed flow cytometry analysis to validate the impact of sleep deprivation on the immune microenvironment in the NSCLC model. Compared with the control group, the proportions of CD3^+^ T and CD8^+^ T cells in the spleen of sleep deprivation group were reduced (Figures 3A and 3B). Meanwhile, the sleep deprivation group exhibited an elevated proportion of CD4^+^ T cells in the tumor tissue, while the proportion of CD8^+^ T cells was significantly reduced (Figures 3A and 3C). Subsequently, we assessed the ratio of NK cells, NKT cells, and Treg cells in both spleen and tumor tissues, and found no statistically significant difference between the control group and the sleep deprivation group (Figures S3A–S3D). Furthermore, we investigated the impact of sleep deprivation on the percentage of macrophages. Flow cytometry analysis indicated that sleep deprivation resulted in elevated macrophage numbers and an increased ratio of M2-polarized to M1-polarized macrophages in both spleen and tumor tissues. (Figures 3D–3F). This suggested that sleep deprivation promoted the polarization of macrophages toward the pro-tumoral phenotype. Collectively, our findings suggested that sleep deprivation induced a shift in the immune cell composition within the NSCLC microenvironment, thereby promoting the establishment of an immunosuppressive milieu.

**Figure 4:**
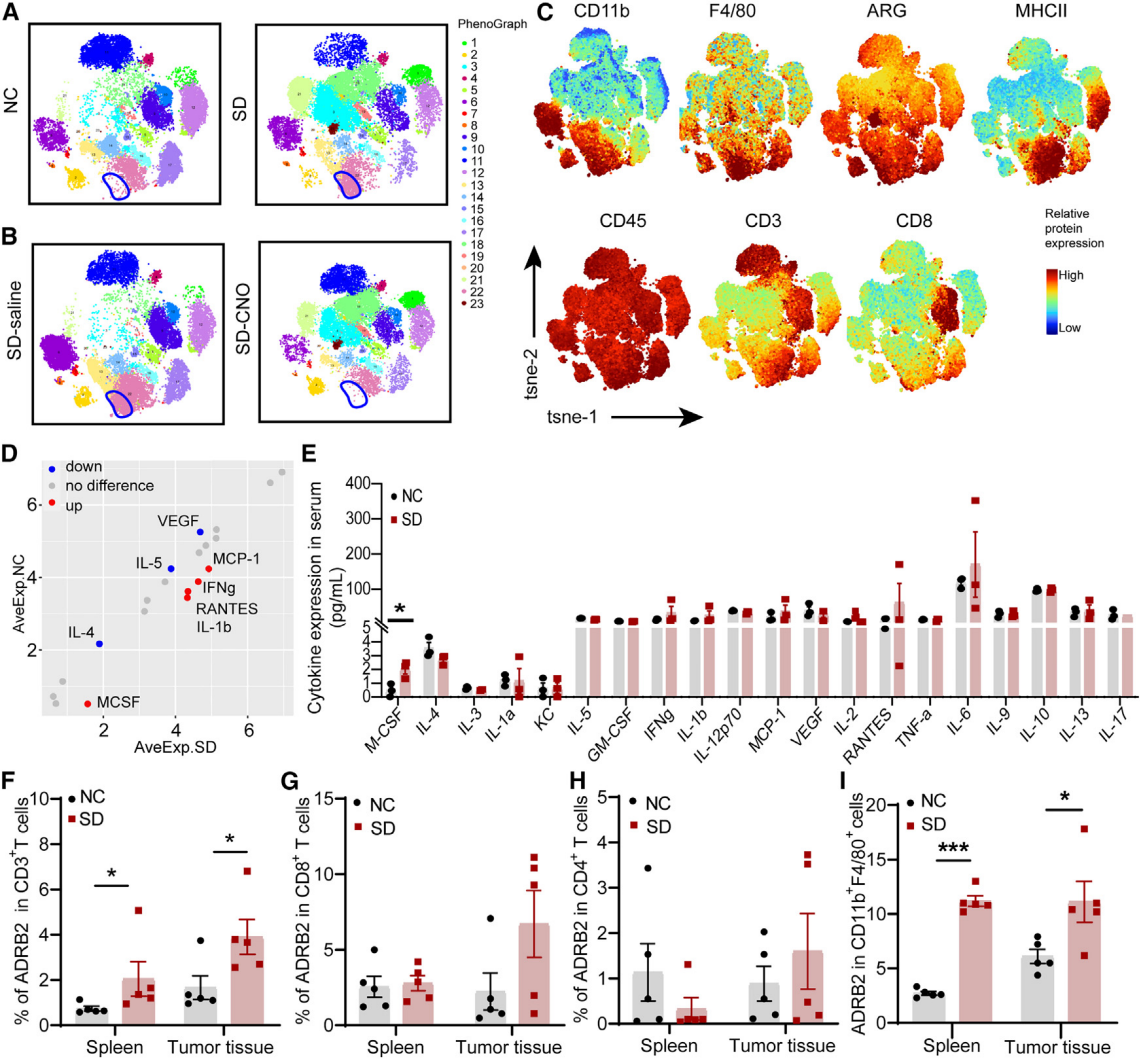
Sleep deprivation affects the expression of ADRB2 on immune cell subsets (A) t-distributed stochastic neighbor embedding (t-SNE) projection of multicolor flow cytometry immunophenotyping of infiltrating immune cells from pulmonary metastasis tumor from NC and sleep deprivation groups. (B) Representative comparison plots of immune cell clusters in PNMT-Cre mice treated with saline or CNO. (C) Display of marker expression from macrophage and T cell in total clusters. (D and E) Detection of differential cytokines in the serum from NC and sleep deprivation groups of tumor-bearing mice (*n* = 3). (F–I) ADRB2 expression on CD3^+^ T, CD8^+^ T, CD4^+^ T cells, and M0 macrophages in spleen and tumor tissue (*n* = 5). Data represented as mean ± SEM. Statistical significance was assessed using Student’s t test or Mann-Whitney U test. ^∗^*p* < 0.05.

Next, we further compared the differences in CD45^+^ immune cell subsets within tumor tissues. Principal-component analysis of flow cytometry mass spectrometry revealed significant difference between the normal sleep group and the sleep deprivation group, indicated by the large disparity between their immune cell subsets. Inhibition of PNMT^RVLM^ neurons of tumor-bearing mice subjected to sleep deprivation reduced the difference with the normal sleep group, suggesting that inhibiting PNMT^RVLM^ neurons may partially mitigate the impact of sleep deprivation on immune cell populations (Figure S4A). Specifically, sleep deprivation led to an increase in the proportion of CD11b^+^F4/80^+^ARG^+^ macrophages (M2 phenotype), a decrease in the proportion of CD11b^+^F4/80^+^MHCII^+^ macrophages (M1 phenotype) and effector CD8^+^ T cells (Figures 4A, 4C, and S4B–S4D). In addition, inhibiting PNMT^RVLM^ neurons during sleep deprivation reduced the proportion of CD11b^+^F4/80^+^ARG^+^ macrophages (Figures 4B and 4C), indicating that inhibition of catecholaminergic neurons in the RVLM might attenuate an immunosuppressive phenotype of macrophages in the sleep-deprived NSCLC mouse model.

Then, we analyzed the expression of cytokines in the serum of tumor-bearing mice in the normal sleep group and sleep deprivation group by cytokine chip array analysis. Compared with the normal sleep group, three cytokines were downregulated and five cytokines were upregulated in the sleep deprivation group (Figure 4D). Notably, there was a statistically significant difference in macrophage colony-stimulating factor (M-CSF) expression between the two groups (Figure 4E). Given the pivotal role of M-CSF in the pro-tumoral macrophage polarization, these findings underscored the contribution of the pro-tumoral phenotype polarization to the sympathetic immune dysfunction induced by sleep deprivation.

### Sleep deprivation-induced ADRB2 activation drives pro-tumor macrophage accumulation and NSCLC progression

**Figure 5:**
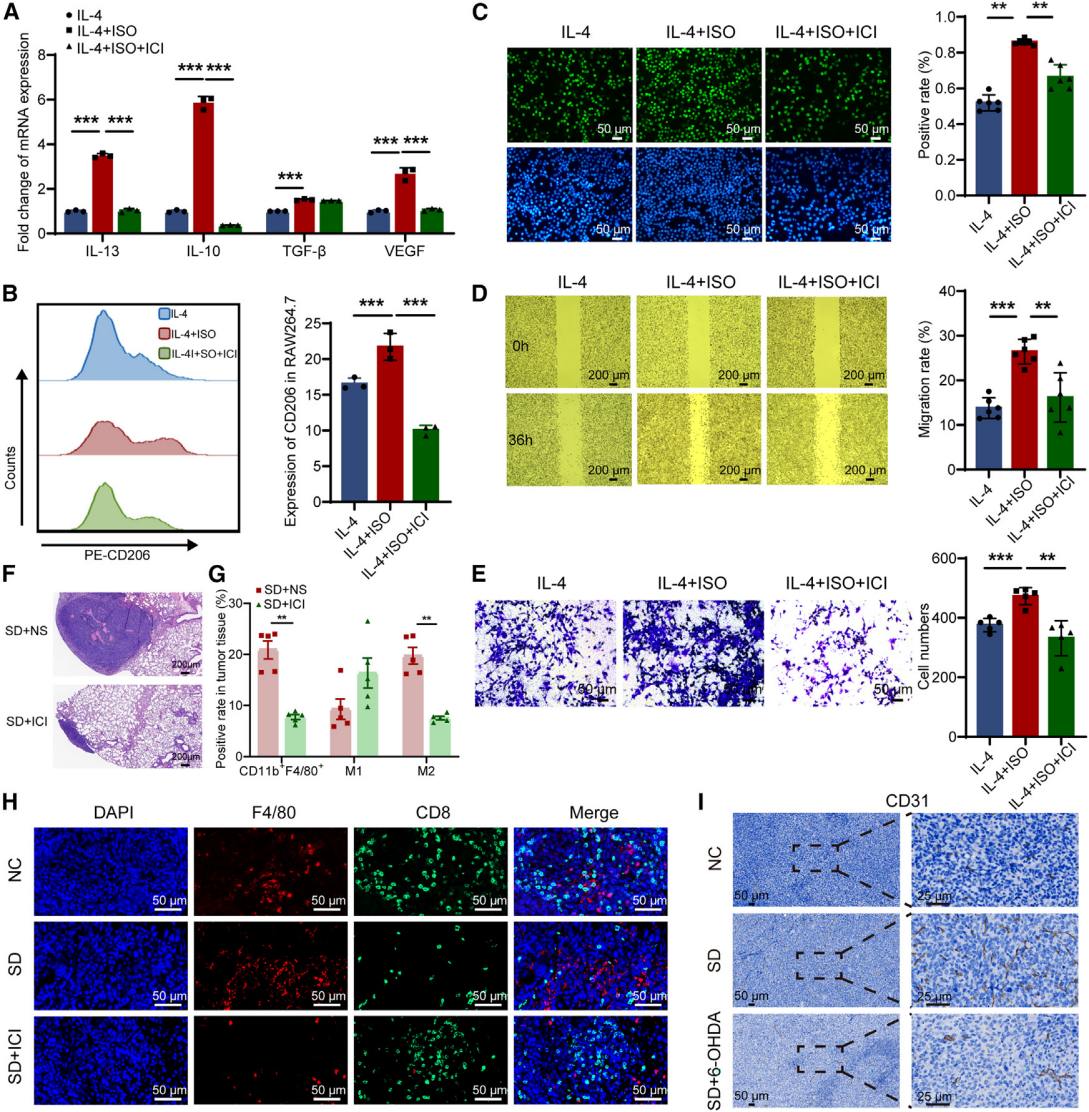
Sleep deprivation induces pro-tumoral phenotype of macrophages through ADRB2 signaling pathways affecting the malignant behavior of NSCLC (A) The effect of ADRB2 on IL-4-induced M2-polarized macrophages-related markers (*n* = 3). (B) The expression of CD206 in macrophages, detected by flow cytometry (*n* = 3). (C) EdU assays of LLC-LUC cells treated with conditioned medium coculture system from supernatant of IL-4-induced macrophages (*n* = 6). (D and E) Wound healing assays and transwell assays of LLC-LUC cells generated by conditioned coculture medium (*n* = 6). (F) Effect of ADRB2 antagonist on tumor metastasis. (G) Proportion of M0 (CD11b^+^F4/80^+^), M1-polarized and M2-polarized macrophages in tumor-bearing mice subject to sleep deprivation after administration of saline or ICI118,551 (*n* = 5). (H) Immunofluorescence images showing the location of F4/80 (red) and CD8 (green) in tumor environment. The nucleus (blue) was stained with DAPI. (I) Representative images of CD31 expression in tumor tissues. Data represented as mean ± SD. Statistical significance was evaluated using Student’s t test. ^∗^*p* < 0.05, ^∗∗^*p* < 0.01, and ^∗∗∗^*p* < 0.001.

The nervous system plays a crucial role in the initiation, progression, and metastasis of tumors, with particular emphasis on its modulation of tumor-associated immune responses, a focal point in cancer neurobiology research.^26^ In order to ascertain the involvement of the sympathetic nervous system in sleep deprivation-induced immunosuppression, we evaluated ADRB2 expression, which links the sympathetic and immune systems, on the surface of immune cell subpopulations within tumor tissues. The results indicated a significant increase in the expression of ADRB2 on CD3^+^ T cells following exposure to sleep deprivation (Figure 4F). While there was an upward trend in ADRB2 expression on CD8^+^ T cells, it did not achieve statistical significance (Figure 4G). Additionally, ADRB2 expression on CD4^+^ T cells showed no statistical difference between the two groups (Figure 4H). It was noteworthy that the expression of ADRB2 of macrophages in the spleen and tumor tissue of the mice in sleep deprivation group was significantly increased (Figure 4I). The results indicated that sleep deprivation-induced activation of the sympathetic nervous system may promote the M2 macrophage polarization through ADRB2 upregulation. To further identify the role of ADRB2 in the M2 macrophage polarization, ADRB2-specific agonist isoprenaline (ISO) was used to stimulate IL-4-induced RAW264.7 cells *in vitro* experiments. The results showed that ISO increased the expression of the pro-tumoral macrophage markers IL-13, IL-10, transforming-growth factor-β (TGF-β), and VEGF. Conversely, ADRB2 antagonist ICI118,551 significantly decreased the expression of IL-13, IL-10, and vascular endothelial growth factor (VEGF) (Figure 5A). In the conditioned media, the secretion of IL-10 and TGF-β by RAW264.7 cells was detected. ISO increased the secretion of both, and adding blockers reduced their secretion levels (Figures S5A and S5B). Furthermore, we utilized flow cytometry to measure the expression of the M2-polarized macrophage marker CD206 in each group. Our findings indicated that the ADRB2 agonist ISO promoted CD206 expression, whereas ICI118,551 inhibited it (Figures 5B and S5C). By evaluating the phagocytic function of macrophages, it was found that ISO inhibited phagocytic function in IL-4-induced macrophages. After adding β2-adrenergic receptor blocker, the phagocytic function was restored (Figure S5D). The findings suggested that ADRB2 played the pivotal role in the induction of pro-tumoral phenotype in macrophages.

To further explore the impact of ADRB2-induced phenotype of macrophages on tumor biology, the supernatant of IL-4-induced RAW264.7 cells was used as the control group. ISO was added to the control group as an ADRB2 stimulant, and ICI118,551 was added as a ADRB2 blockade group. The supernatants of the three groups were used as conditioned media for co-culture with LLC-LUC cells. EdU proliferation experiment showed that compared with the control group, the supernatant of ISO stimulation group promoted the proliferation of LLC-LUC cells, while ICI118,551 inhibited their proliferation (Figure 5C). Wound healing experiment showed that culture supernatant supplemented with ISO stimulation promoted the migration of LLC-LUC cells, while ICI118,551 inhibited their migration (Figure 5D). Similarly, transwell experiment showed that the coculture supernatant added with ISO promoted the invasion ability of LLC-LUC cells, while the inhibitor hindered their invasion (Figure 5E). These results demonstrated that activating ADRB2 promoted polarization of macrophages toward the pro-tumoral phenotype and thereby promoting the proliferation, migration, and invasion of LLC-LUC cells.

Furthermore, we validated the regulatory role of ADRB2 *in vivo* by administering ICI118,551 to sleep-deprived tumor-bearing mice, inhibiting NSCLC development (Figure 5F). Particularly, flow cytometry analysis of macrophage phenotypes in tumor tissue revealed that sleep-deprived mice treated with ICI118,551 had a lower total macrophage count and a reduced proportion of M2-polarized macrophage infiltration compared to the saline treatment group (Figure 5G). The results showed that the proportion of F4/80^+^ macrophages and the expression of CD31, surface marker of neovascular endothelial cells, in the tumor tissue of sleep deprived mice were significantly increased, while the proportion of CD8^+^ T cells was markedly decreased. Moreover, macrophages predominantly localized within the tumor core, whereas CD8^+^ T cells were predominantly found surrounding the tumor periphery, suggesting that the macrophages induced by sleep deprivation might form a physical barrier to inhibit CD8^+^ T cells infiltration into the tumor. Meanwhile, ICI118,551 inhibited macrophages infiltration and CD31 expression while facilitating infiltration of CD8^+^ T cells (Figures 5H and 5I). In summary, our experiments indicated that ADRB2-mediated pro-tumoral macrophage, induced by sleep deprivation, inhibited CD8^+^ T cells infiltration and promoted the proliferation, migration, and invasion of NSCLC *in vivo* and *in vitro*.

### KLF4 mediates ADRB2-induced pro-tumoral macrophage polarization

**Figure 6:**
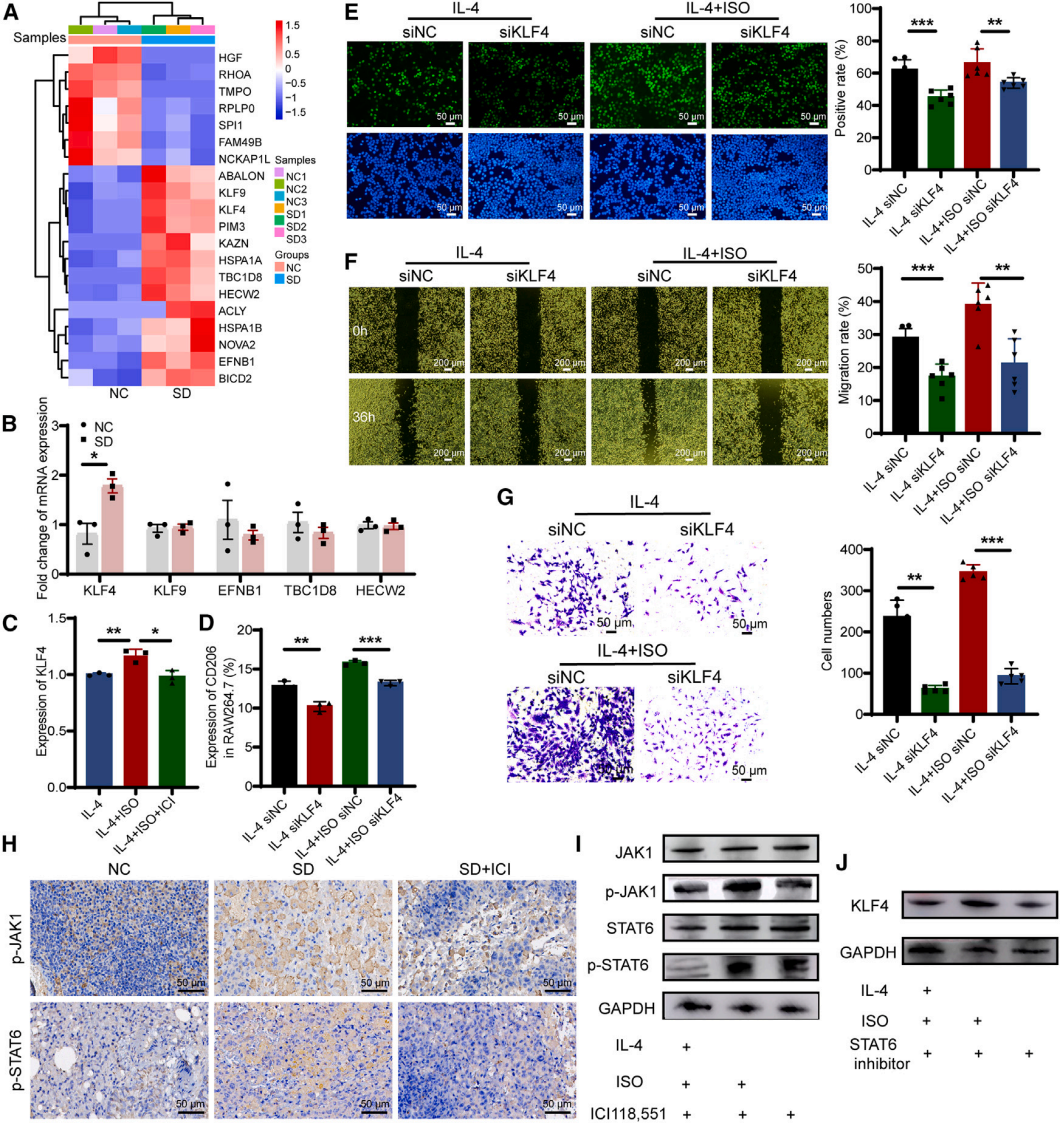
KLF4 is associated with sleep deprivation-induced ADRB2 modulation of macrophage prompting on proliferation, migration, and invasion of LLC-LUC cells (A) Differentially expressed Top20 genes in metastatic lung tissues of NC group and sleep deprivation group. (B) The expression of top 5 upregulated genes in tumor tissues was analyzed by qRT-PCR. (C) KLF4 expression was detected by ADRB2-specific agonist ISO and ADRB2 antagonist ICI118,551 in macrophages treated with IL-4 *in vitro*. (D) The effect of ADRB2 agonist on CD206 expression in IL-4-induced KLF4-knockdown macrophages. (E) EdU assays showed the proliferation of tumor cells in the coculture system with 30% CM. CM derived from IL-4 and IL-4 + ISO induced supernatant of macrophages with KLF4 low and high expression. (F) The migration ability of tumor cells was determined by wound healing assays in the indicated coculture medium. (G) Transwell assays demonstrated invasion of tumor cells in the indicated coculture system. (H) Representative IHC images of p-JAK1 and p-STAT6 in xenograft tumors. (I) The phosphorylation of JAK1 and STAT6 expression in RAW264.7 treated with ADRB2 agonist or antagonist via Western blotting. (J) Immunoblotting of KLF4 in RAW264.7 treated with different conditions. Data represented as mean ± SD. Statistical analysis was performed using Student’s t test or Mann-Whitney U test. ^∗^*p* < 0.05, ^∗∗^*p* < 0.01, and ^∗∗∗^*p* < 0.001.

To further elucidate the molecular regulatory network underlying sleep deprivation-induced promotion of NSCLC metastasis, transcriptome sequencing was performed in the normal sleep group and sleep deprivation group. Among the differentially expressed genes of top 20, there were 13 upregulated genes and 7 downregulated genes (Figure 6A). Subsequent qPCR results about the top five genes among the upregulated genes showed that sleep deprivation increased KLF4 expression (Figure 6B). Experiments *in vitro* showed that ISO stimulation could increase KLF4 expression in RAW264.7 cells, while ADRB2 antagonist ICI118,551 inhibited KLF4 expression (Figure 6C). *In vivo* experiments demonstrated that, compared to the normal sleep group, sleep deprivation exhibited elevated levels of KLF4 expression, whereas the ICI118,551 treatment group showed a decrease in KLF4 expression (Figure S6A). However, ADRB2 stimulation had no significant effect on KLF4 expression in LLC cells (Figure S6B), suggesting that sleep deprivation-induced ADRB2 functions through KLF4 in macrophages.

To further clarify whether KLF4 affected the M2-phenotype polarization of RAW264.7, KLF4 was knocked down using RNA small interfering technology (Figure S6C). The flow cytometry results showed that the expression of CD206 in siKLF4 group was significantly lower compared to the siNC group under treatment of IL-4. Moreover, costimulation test with IL-4 and ISO demonstrated that KLF4 knockdown significantly inhibited CD206 and VEGF expression. In addition, knockdown of KLF4 inhibited the enhancement of CD206 expression in RAW264.7 cells following ADRB2 activation (Figures 6D and S6D). These findings suggested that KLF4 may be involved in the M2 macrophage polarization induced via ADRB2 during sleep deprivation.

To further elucidate whether KLF4 expression in RAW264.7 cells affects NSCLC biological functions, the supernatants from stimulated RAW264.7 cells were utilized as conditioned media cocultured with LLC-LUC cells. When stimulated solely with IL-4, the supernatant from KLF4-knockdown cells co-cultured with LLC-LUC suppressed its proliferation capability. Likewise, in the presence of both IL-4 and ISO stimulation, the supernatant from KLF4-knockdown cells co-cultured with LLC-LUC similarly hindered its proliferation capacity (Figure 6E). In cell migration assays, the supernatant from KLF4-knockdown cells co-cultured with LLC-LUC cells inhibited its migration ability (Figure 6F). In transwell invasion assays, the co-culture system involving KLF4-knockdown cells significantly inhibited the invasion of LLC-LUC cells. (Figure 6G). These results suggested that knocking down KLF4 in the RAW264.7 cells inhibited proliferation, migration, and invasion abilities of NSCLC, indicating that KLF4 might be involved in sleep deprivation-promoted NSCLC progression. The regulatory mechanism of KLF4 involved in sleep deprivation remains unclear, despite its crucial role in the pro-tumoral phenotype of macrophages. A pathway enrichment analysis was conducted on the differential cytokines in the serum of normal sleep and sleep-deprived tumor-bearing mice, revealing a significant enrichment of STAT-related pathways (Figure S6E). Results showed that the expression levels of p-JAK1 and p-STAT6 in the tumor tissue of the sleep deprivation group were significantly upregulated, while ICI118,551 inhibited the expression of p-JAK1 and p-STAT6 (Figures 6H and 6I). These findings indicate that the ADRB2 modulates the phosphorylation of the JAK1-STAT6 signaling pathway. The use of the STAT6 inhibitor AS1517499 demonstrated an inhibition of ISO-mediated enhancement of KLF4 expression (Figure 6J). Collectively, these findings suggested that the JAK1-STAT6 pathway might be involved in promoting the pro-tumoral macrophage polarization via ADRB2/KLF4 in response to sleep deprivation, thereby facilitating the progression of NSCLC.

### Sleep deprivation elevates NE and links ADRB2 to poor prognosis in NSCLC

**Figure 7:**
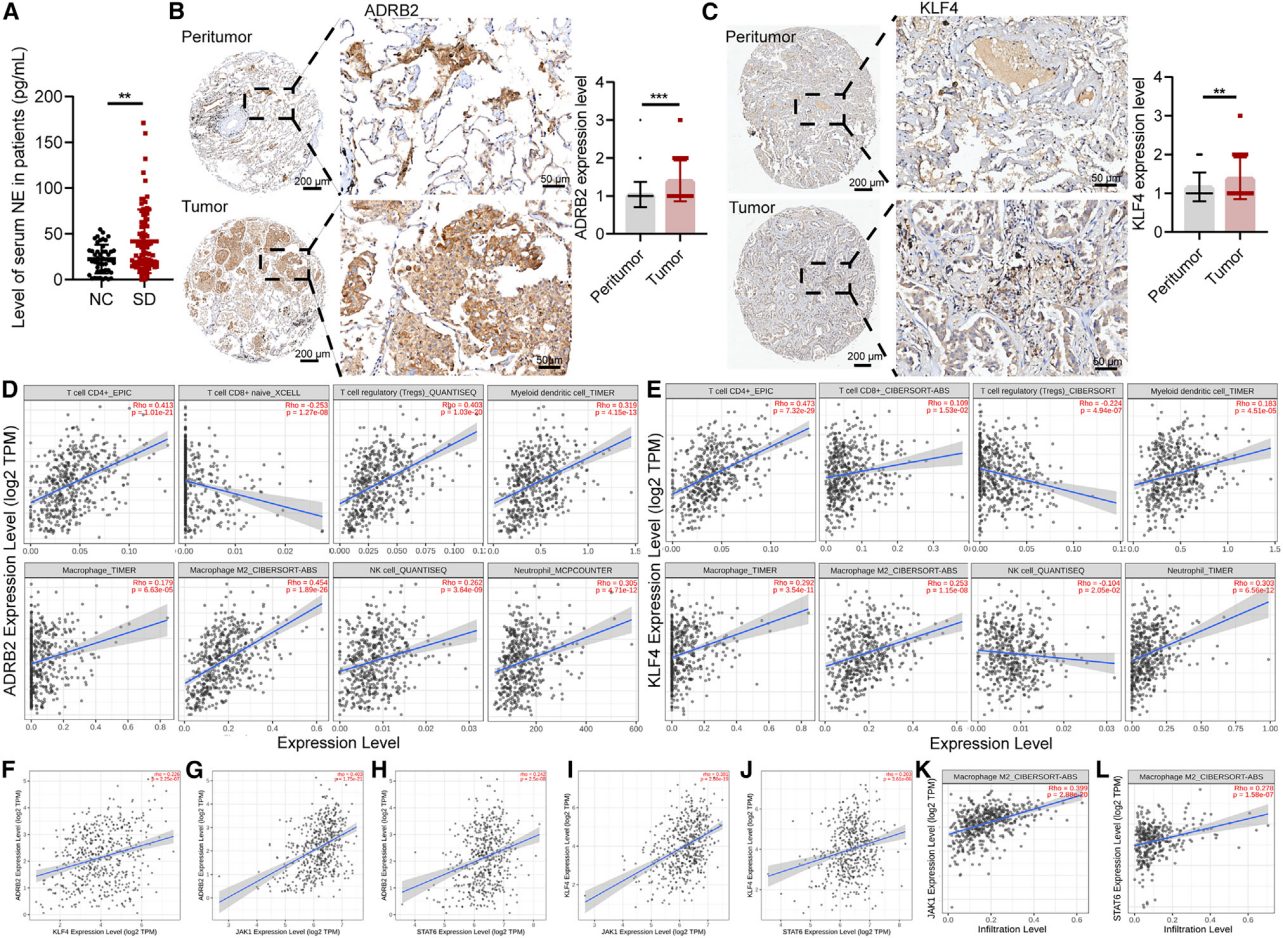
NE level is elevated in NSCLC patients with sleep deprivation and is associated with a poor prognosis (A) The concentration of NE in serum of the NSCLC patients, determined by ELISA assay. (B) Representative IHC images and quantitative analysis of ADRB2 in the NSCLC patients. (C) Representative IHC images and quantitative analysis of KLF4 in the NSCLC patients. (D) Investigation into the association between ADRB2 expression and immune cell infiltration in patients diagnosed with LUAD. (E) Analysis of the correlation between KLF4 and immune cell infiltration in LUAD patients. (F–H) Correlation analysis between ADRB2, KLF4, JAK1, and STAT6 expression. (I and J) Correlation analysis between KLF4, JAK1, and STAT6 expression. (K and L) Correlation analysis between JAK1, STAT6, and pro-tumoral macrophages. Data represented as mean ± SD. Statistical analysis was evaluated using Student’s t test. ^∗∗^*p* < 0.01 and ^∗∗∗^*p* < 0.001.

To elucidate the clinical significance of sleep deprivation in NSCLC, we conducted an analysis of sleep patterns using a questionnaire administered to 131 NSCLC patients admitted to our hospital. The results showed that 64.12% (*n* = 47) of the NSCLC patients had less than 6 h of nightly sleep. NSCLC patients were stratified into a sleep deprivation group (*n* = 84) and a normal sleep duration group (*n* = 47). Serum samples were collected from both cohorts to assess NE expression. The results showed that NE expression was significantly higher in the sleep deprivation group than in the NC group (Figure 7A), suggesting a potential link between sleep deprivation and NE induction. Additionally, immunohistochemical experiments were performed on tissue chips to evaluate ADRB2 and KLF4 expression levels in tumor tissues of NSCLC, revealing markedly higher expression levels compared to adjacent normal tissues (Figures 7B and 7C). Clinical analysis further indicated that high ADRB2 expression was closely associated with advanced clinical stage in NSCLC patients (Table S1). Furthermore, we investigated the association between ADRB2 and KLF4 in immune cell infiltration among lung cancer patients by leveraging the TIMER2.0 database. The results showed that ADRB2 and KLF4 were significantly correlated with CD4^+^ T cells, CD8^+^ T cells, macrophages, NK cells, and neutrophils infiltration in lung adenocarcinoma (LUAD), notably both showing a positive correlation with the pro-tumoral macrophages. (Figures 7D and 7E). Furthermore, we also found that ADRB2 was significantly positively correlated with the expression levels of KLF4, JAK1, and STAT6 (Figures 7F –7H). Meanwhile, we found that KLF4 was positively correlated with the expressions of JAK1 and STAT6 (Figures 7I and 7J). Besides, we found that the infiltration of M2-polarized macrophages was correlated with JAK1 and STAT6 in LUAD (Figures 7K–7L). These findings highlighted the potential of ADRB2 expression as a prognostic indicator for NSCLC and provided evidence for its potential role in regulation of KLF4 through JAK1/STAT6 pathway. However, further mechanistic investigations were warranted.

## Discussion

The present study demonstrated that sleep deprivation facilitated the progression and metastasis of NSCLC. Mechanistic research revealed that sympathetic activation induced by sleep deprivation promoted the polarization of macrophages to the pro-tumoral phenotype via the ADRB2/KLF4 pathway, mediated by JAK1/STAT6 phosphorylation. Our findings provided direct experimental evidence and proposed a mechanistic foundation for the detrimental effects of sleep deprivation on NSCLC progression, highlighting the interaction between the neuroscience and immune system.

Sleep deprivation, a classical chronic stress, has multiple effects on the nervous system.^27^ Studies have found that stress stimulates the hypothalamic-pituitary-adrenal (HPA) axis and the sympathetic nervous system, leading to abnormal hormone release, which promotes cancer biological processes through receptors, including proliferation, genomic instability, angiogenesis, metastasis, immune evasion, and metabolic dysregulation.^28^ Our study revealed that sleep deprivation increased the activity of peripheral and central sympathetic nervous. Chronic activation of the sympathetic nervous system is considered a pivotal factor associated with poor prognosis in cancer patients.^29^ Sympathetic activity, which causes an increase in adrenergic signaling, affects the shape, maturation, and releases of immune cells into the bloodstream, and non-selective blockade of beta receptors can inhibit stress-induced activation of neurons and corresponding activation in the endocrine and immune systems.^30^ For instance, patients with oral precancerous lesions (leukoplakia) showed increased plasma NE levels and a highly active sympathetic nervous system after sleep deprivation.^31^ A study on the liver sympathetic nervous system showed that sleep deprivation promoted liver steatosis by increasing intrahepatic nerve growth factor and NE, increasing the risk of liver cancer,^32^ indicating that the sympathetic nervous system is involved in sleep deprivation promoting tumor development. In this study, we constructed subcutaneous tumor models and metastasis models of NSCLC and subjected them to sleep deprivation, and found that sleep deprivation facilitated the progression and metastasis of NSCLC by activating the sympathetic nervous system. Furthermore, NE level was elevated in the serum of NSCLC patients experiencing sleep deprivation, and the higher expression of ADRB2 was associated with a poor prognosis in clinical study.

Numerous studies have revealed the closely intertwined relationship between the nervous system and the immune system. Huang et al. found that the lymph nodes are governed by sensory neurons situated in the dorsal root ganglion and form a sensory neuro-immune circuit to sustain lymph-borne inflammatory signals.^33^ Zhang et al. found that activation of the catecholaminergic neurons in the VLM decreased CD8^+^ T cells to promote tumor progression.^17^ The RVLM is the central regulator of sympathetic nerve activity, and its efferent fibers connect with preganglionic sympathetic neurons in the spinal cord to form a monosynaptic connection. Postganglionic fibers then innervate the adrenal glands, lymphoid tissues and lungs, regulating peripheral sympathetic nerve activity. Our study demonstrated that sleep deprivation increased the excitability of catecholaminergic neurons in the RVLM, thereby stimulating the sympathetic nervous system. Chemogenetics inhibition of these neurons neutralized NSCLC metastasis, indicating that the RVLM played an important role in promoting NSCLC metastasis during sleep deprivation. There is compelling evidence indicating that the nervous system can significantly impact the prognosis of various types of cancer, and the interplay between cancer and the nervous system has emerged as a pivotal focus in biomedical research.^34^ Mapping out a network of interactions between tumors and center neural system in the brain holds great potential for advancing tumor neuro-immunology research.

In our study, we found that NE was the predominant upregulated neurotransmitter of sleep deprivation-activated sympathetic nervous system. Numerous studies reveal that NE exerts immunosuppressive effects by altering the expressions of some immune-related genes in dendritic cells (DCs), macrophages, and myeloid-derived suppressor cells (MDSCs), such as iNOS2, Arg1, and IL-10.^35^ β-adrenergic receptor antagonist propranolol effectively abolishes this neuroimmune effect, confirming that sympathetic nerve signals directly enhance the immunosuppressive function of MDSCs.^36^ Besides, T cells, NK cells, and macrophages in tumor microenvironment mainly express ADRB2 on their surfaces. Sympathetic nervous system activation leads to an increase in catecholamine neurotransmitters, which is associated with tumor progression, and may be related to catecholamine-induced M2 polarization of macrophages.^37^ In this study, we found that sleep deprivation remodeled the tumor immune microenvironment to promote metastasis of NSCLC. There is increasing evidence that neural and immune factors play important roles in the coordinated regulation of the tumor microenvironment.^16^^,^^23^^,^^38^ In the present study, we conducted flow cytometry to detect the expression of ADRB2 on immune cells, which is a key receptor for studying sympathetic-immune dysregulation and promoting tumor growth and metastasis through various mechanisms. The results showed that sleep deprivation promoted the expression of ADRB2 on CD3^+^ T cells in the spleen and tumor tissues. In addition, sleep deprivation resulted in a reduction of CD3^+^ T cells in the spleen and an increase in ADRB2 expression, accompanied by a decrease in the proportion of CD8^+^ T cells both in the spleen and tumor tissues. These findings suggested that sleep deprivation might activate sympathetic activity innervating the spleen, which in turn acted on ADRB2 to diminish CD8^+^ T cells. Future research will aim to explore this phenomenon in greater depth.

Our further analysis demonstrated that sleep deprivation significantly increased the population of macrophages in both the spleen and tumor tissue. Specifically, it enhanced the proportion of pro-tumoral macrophages while reducing the proportion of anti-tumoral macrophages, thereby promoting tumor angiogenesis and upregulating the expression of ADRB2 on macrophages. An increased proportion of M2 macrophages and a decreased proportion of CD8^+^ T cells in tumor tissues, resulting in the formation of a tumor immune-suppressive microenvironment. Macrophages are one of the most important cells in the innate immune system, and M0 macrophages show significant plasticity in response to different microenvironmental stimuli, which can be converted into two subtypes with different molecular signatures and functional characteristics, including the classically activated M1 anti-tumoral phenotype and the alternatively activated M2 pro-tumoral phenotype.^39^ M2 macrophages are generally considered to be tumor-associated macrophages and are the main immune cell population in tumor microenvironment, participating in immune suppression, neovascularization, metastasis, and resistance to immunotherapy, and able to secrete various cytokines to promote tumor progression.^39^^,^^40^^,^^41^ The maintenance of homeostasis in various tissues requires neural-immune communication between the sympathetic nervous system and immune cells, and the sympathetic nervous system regulates the anti-inflammatory state of macrophages in adipose tissue through the noradrenergic pathway.^42^ In a study on the mechanism of accelerating fracture healing in traumatic brain injury, it was found that noradrenergic signal regulation of M2 macrophage infiltration played a role in the healing tissue, and the addition of ADRB2 agonists accelerated tissue healing,^43^ indicating that the ADRB2 signal had a regulatory role in the polarization of M2 macrophages. In this study, we found that blocking ADRB2 reduced the number of macrophages in tumor tissue and decreased the proportion of M2-polarized macrophages. The ADRB2 within the sympathetic pathway has been implicated in facilitating the infiltration of macrophages and promoting their M2 pro-tumoral phenotype within spleen and tumor tissues. This mechanism has been suggested to contribute to the progression of NSCLC under conditions of sleep deprivation.

KLF4 is an environmentally dependent transcription factor that acts as a tumor suppressor or oncogene, exerting anti-cancer effects by increasing cell proliferation, metabolism, and reducing ROS through the molecular pathway of glycolysis.^44^ KLF4 serves as an important transcription factor in the polarization process of macrophage, and studies have shown that KLF4 and STAT6 promote M2 polarization and inhibit M1 polarization. Our research revealed that by inhibiting the expression of KLF4, the promotion of pro-tumoral macrophage polarization and the malignant biological behavior of NSCLC induced by ADRB2 agonists could be nullified. KLF4, a key regulator of the STAT6 signaling pathway, plays a crucial role in macrophage polarization.^45^^,^^46^ JAK1-STAT6 pathway is one of the key pathways involved in the formation of pro-tumoral macrophages, and STAT6 has a dual role as a transcription factor and a signaling molecule, which is closely related to the IL-13/IL-4 signaling pathway. When stimulated by IL-4 or IL-13, STAT6 binds to the corresponding receptor, undergoes phosphorylation, dissociation, dimerization, and then enters the nucleus to regulate the expression of M2-phenotype-correlated genes in macrophages. The results showed that the expression of KLF4, p-JAK1, and p-STAT6 was increased in the tumor tissue of the sleep deprivation group compared to the normal sleep group. Additionally, there was an increase in the proportion of macrophages and pro-tumoral macrophages in the tumor microenvironment. Conversely, upon administration of ADRB2 blockers, the expression level of KLF4, p-JAK1, and p-STAT6 was reduced, along with a decrease in the proportion of pro-tumoral macrophages in the tumor microenvironment. The activation of ADRB2 of RAW264.7 led to increased expression of p-JAK1 and p-STAT6, while the suppression of ADRB2 resulted in diminished expression of p-JAK1 and p-STAT6. These findings suggested that the activation of ADRB2 promoted the phosphorylation of JAK1 and STAT6 as well as KLF4 expression. Furthermore, the addition of a STAT6 phosphorylation inhibitor reduced KLF4 expression, indicating that the ADRB2 might promote KLF4 expression through the phosphorylation of the JAK1-STAT6 pathway.

The results from the TIMER2.0 database demonstrated that ADRB2 expression was inversely associated with CD8^+^ T cell infiltration, while it showed a positive association with both the overall macrophage population and pro-tumoral macrophage infiltration. Likewise, KLF4 expression was positively correlated with macrophage numbers and pro-tumoral macrophage infiltration. Additionally, ADRB2 was positively correlated with JAK1/STAT6/KLF4 expression. These database results further suggested that the ADRB2/KLF4 pathway was involved in macrophage M2 polarization. These findings suggest that sleep deprivation activates the JAK1-STAT6 pathway through ADRB2, thereby promoting the pro-tumoral phenotype of macrophages in NSCLC. However, the precise regulatory mechanism by which ADRB2 modulates KLF4 expression via JAK1/STAT6 necessitates further investigation.

In summary, activation of the sympathetic nervous system induced by sleep deprivation facilitates macrophages’ polarization to the pro-tumoral phenotype via the ADRB2/KLF4 pathway. Targeting ADRB2 has the potential to impede the progression of NSCLC induced by sleep deprivation (Graphical abstract).

### Limitations of the study

This study has delved into the mechanism by which sleep deprivation facilitates the metastasis of NSCLC through the activation of both the peripheral and central sympathetic nervous systems. In this process, the RVLM, acting as a sympathetic center, plays a pivotal regulatory role. However, the specific neural circuit mechanisms by which sleep deprivation modulates RVLM excitability remain poorly understood and warrant further in-depth investigation.

## Resource availability

### Lead contact

Further information and requests for resources and reagents should be directed to and will be fulfilled by the lead contact, Lihua Liu (cdlihualiu@hebmu.edu.cn).

### Materials availability

This study did not generate new unique reagents.

### Data and code availability

Transcriptome sequencing data have been deposited at Gene Expression Omnibus (GEO) as GSE274724, GSE274725 and are publicly available as of the date of publication. This paper does not report original code. Any additional information required to reanalyze the data reported in this paper is available from the lead contact upon request.

## Acknowledgments

We thank Huixian Cui at International Cooperation Laboratory of Stem Cell Research, Hebei Medical University, China, for his kindly assistance. This work was supported by the 10.13039/501100001809National Natural Science Foundation of China (81871894 and 82203079), the Clinical Research and Innovation Team Support Program of 10.13039/501100012505Hebei Medical University (2022LCTD-A11), the Research and Innovation Team Support Program of Fourth Hospital of Hebei Medical University (2023C11), the Hebei Province Graduate Innovation Funding Project (XCXZZB202313).

## Author contributions

L.L. and S.W. conceived and designed the research; S.Y., J.W., Y.J., X.W., Y.Z., T.L., W.L., Y.D., and S.Z. performed the experiments and analyzed the data; S.Y., J.W., and Y.J. prepared the manuscript. S.W. and L.L. provided critical revisions for important intellectual content within the manuscript. All authors read and approved the final manuscript.

## Declaration of interests

The authors declare no competing interests.

## STAR★Methods

### Key resources table

**Table:** 

REAGENT or RESOURCE	SOURCE	IDENTIFIER
**Antibodies**		

anti-cFos	Abcam	Cat#ab214672; RRID: AB_2939046
anti-TH	Abcam	Cat#ab137869; RRID: AB_2801410
anti-CD3	Tonbo	Cat#35-0032; RRID: AB_2621660
anti-CD4	Tonbo	Cat#25-0041; RRID: AB_2904484
anti-CD8	Tonbo	Cat#67-0081
anti-CD25	Tonbo	Cat#20-0251; RRID: AB_2621567
anti-NK1.1	Tonbo	Cat#25-5941
anti-CD45	BD	Cat#557659; RRID: AB_396774
anti-CD11b	Tonbo	Cat#35-0112; RRID: AB_2621676
anti-CD86	Tonbo	Cat#60-0862
anti-F4/80	Elabscience	Cat#E-AB-F0995I
anti-CD206	Biolegend	Cat#141708; RRID: AB_10900231
anti- JAK1	Proteintech	Cat#66466-1-Ig; RRID: AB_2881834
anti- pJAK1	ImmunoWay	Cat#YP0154
anti- STAT6	Proteintech	Cat#66717-1-Ig; RRID: AB_2882068
anti- pSTAT6	ImmunoWay	Cat# YP0256
anti-KLF4	Proteintech	Cat#11880-1-AP; RRID: AB_10640807
anti-ADRB2	biorbyt	Cat#Orb495085

**Chemicals, peptides, and recombinant proteins**		

IL-4	ABclonal	RP01161
isoprenaline	Cayman	Cat#15592
ICI118,551	GLPBIO	Cat#72795-01-8
AS1517499	MCE	Cat#HY-100614
D-Luciferin (potassium salt)	Cayman	Cat#14681
6-hydroxydopamine	Cayman	Cat#25330
PD-1 inhibitor	Bio X Cell	Cat#BP0273
clozapine N-oxide	Tocris	Cat#6329/10

**Critical commercial assays**		

mouse cytokine array	RayBiotech	QAM-CYT-1
siRNA/miRNA transfection reagent	Yeasen	Cat#40806ES02
NE ELISA kits (mouse)	Arigo Biolaboratories	ARG80475
NE ELISA kits (human)	CUSABIO	CSB-E07021h
Cytofix/Cytoperm kit	BD	554714
TGF-β ELISA kits (mouse)	Bioswamp	MU30071
IL-10 ELISA kits (mouse)	Bioswamp	MU30055
Vybrant TM phagocytosis assay kit	Molecular Probes	V6694

**Deposited data**		

Transcriptome sequencing (tumor tissue)	GEO	GSE274724
Transcriptome sequencing (RAW264.7)	GEO	GSE274725

**Experimental models: Cell lines**		

RAW264.7	ScienCell	N/A
LLC-LUC	ScienCell	N/A

**Software and algorithms**		

CytExpert	Beckman	https://www.mybeckman.cn
LabChart 8	ADInstruments	https://www.adinstruments.com/support/downloads/windows/labchart-chinese
FlowJo-v10.8.1	FlowJo LLC	https://flowjo.bectondickinson.cn
GraphPad Prism v8.0.2	GraphPad Software	https://www.graphpad.com

### Experimental model and subject participant details

PNMT-Cre mice and AAV-hSyn-DIO-hM4Di-EGFP virus were generously gifted by Prof. Sheng Wang (Hebei Key Laboratory of Neurophysiology, Hebei Medical University). The C57BL/6 mice utilized in this investigation were procured from Beijing Vital River Laboratory Animal Technology Co., Ltd. Mice of identical age (6-8 weeks) and gender (regardless of male or female) were randomly assigned to experimental cohorts and maintained under specific pathogen-free (SPF) conditions. The experimental protocol was approved by the Animal Care and Ethics Committee of the Fourth Hospital of Hebei Medical University (IACUC-4th Hos Hebmu-20240203). This study used a commercial tissue microarray chip whose preparation was approved by the Research Ethics Committee of People’s Hospital of Tongxu (ZL-XP201402). All patients providing clinical specimens signed the written informed consent form. All procedures were carried out in accordance with the principles of Declaration of Helsinki.

### Method details

#### Xenograft tumor mouse model

1 × 10^6^/100 μL Lewis lung carcinoma harboring luciferase (LLC-LUC) cells were injected subcutaneously into the right inguinal region of C57BL/6J mice, and the mice were divided into sleep deprivation group and regular sleep group 3 days after inoculation. Length (L) and width (W) of the tumors were measured per 3 days using a vernier caliper, and the tumor volume (V) was calculated according to the formula: V = (π/6) × L × W^2^. After 14 days of sleep deprivation, mice were anesthetized with pentobarbital sodium (60 μg/g, i.p.), and blood was collected from the orbital vein. The tumor tissues were harvested and their weights were measured.

C57BL/6J mice were injected with 5 × 10^5^/100 μL LLC-LUC cells into the tail vein to establish a lung metastasis tumor model, and after 21 days of sleep deprivation, fluorescein potassium salt (150 mg/kg, Cayman, USA) was injected into the abdominal cavity. After 10 min of intraperitoneal injection of fluorescein in mice, the IVIS Spectrum CT Imaging System (PerkinElmer, USA) was used to take pictures and analyze the fluorescence intensity to evaluate the progress of the tumor. Blood, spleen, and tumor tissues were collected for immunohistochemistry (IHC) and flow cytometry.

#### Sleep deprivation mouse model

To eliminate the interference of curiosity, exploration, etc., all mice were placed on Pinnacle sleep deprivation instruments for 6 h/day for 3 consecutive days to adapt to the new environment. The mice were placed on an automatic moving horizontal bar to induce sleep deprivation, and they were awakened by the rotating bar as soon as they fell asleep to achieve sleep deprivation. As control, the normal sleep group was placed in the same environment but without the rotating horizontal bar so that they could continue sleeping. During this period, the mice were free to move around and had access to food and water. After subcutaneous injection of LLC-LUC cells into C57BL/6J mice for 3 days, they were randomly divided into the normal sleep group and the sleep deprivation group. The sleep deprivation group was deprived of sleep from 11:00 a.m. to 5 p.m. per day for 2 weeks.

After a 3-day period following intravenous injection into the tail vein of LLC-LUC cells, C57BL/6J mice were deprived of sleep for 6 h/day for 3 weeks. All mice were housed under the same 12h light/dark cycle, ambient room temperature as well as with food and water available *ad libitum* in specific pathogen-free conditions.

#### HE staining

First, the mouse tumor tissues were fixed with 4% paraformaldehyde. Then, they were dehydrated with alcohol and embedded in paraffin, cut into 5 μm thick sections, dewaxed, hydrated, and washed with double distilled water. Next, the tissue sections were stained with hematoxylin for 8 min and rinsed in water. Then, they were immersed in 1% acidic alcohol for 5 min and then rinsed with running water. Additionally, the tissue sections were stained with eosin for 3 min, dehydrated, and cleared with xylene. Finally, the sections were mounted with mounting medium and the stained samples were observed under an optical microscope.

#### Polysomnographic recordings

The mice were anesthetized with pentobarbital sodium (60 μg/g, i.p.), and their head and neck hair were shaved. They were then secured on a stereotaxic apparatus to fully expose the fontanels. The electrodes for recording the mice's brain electrical activity were implanted, and a drill hole was drilled through the skull to reach the depth where the screws just touched the cortex. Subsequently, the electrodes for gathering muscle electrical activity were inserted into the neck muscles and secured with sutures. These electrodes were affixed to the skull using a mixture of dental acrylic resin and monomer, followed by suturing the incision. After 7 days of the surgery for recovery, EEG/EMG data was captured and analyzed with Sirenia sleep software, whereby the signals were amplified, filtered, and converted to digital format (EEG, 5 - 30 Hz; EMG, 40 - 200 Hz) to monitor sleep states. The software automatically distinguished between wakefulness (W), non-rapid eye movement (NREM) and rapid eye movement (REM) sleep (wake: desynchronized EEG patterns accompanied by high EMG activity. NREM: synchronized EEG exhibiting high amplitude, low-frequency activity (0.5 - 4 Hz) and minimal EMG activity. REM: prominent power in theta frequencies (4 - 9 Hz) in the EEG coupled with low EMG activity). Manual adjudication was employed as necessary for correction.

#### Transcriptome sequencing

First, 1 μg total RNA was dissolved in nuclease-free H_2_O to a total volume of 50 μL in nuclease-free PCR tubes and stored at -20°C. The mRNA capture beads were used to separate mRNA from fragmentation. Double-stranded cDNA was synthesized and then purified cDNA using magnetic beads. End repair and end dA-Tailing was conducted. RNA adapters were added to the sample for the connection reaction. VAHTS DNA clean beads were added to the connected product to bind cDNA to magnetic beads for product purification and fragment size selection. The PCR Primer Mix and Amplification Mix 1 were used to amplify the library. The PCR products were purified using magnetic beads. The library was quantified for preparing chips. The chip was placed in the analyzer and verified.

#### Enzyme-linked immunosorbent assay (ELISA)

Norepinephrine (NE) in the serum of tumor-bearing mice and NSCLC patients were measured using ELISA kits (mNE: Arigo Biolaboratories Co., Shanghai, China. hNE: CUSABIO, Wuhan, China) following the manufacturer’s instructions.

The levels of TGF-β and IL-10 in the conditioned media of Raw264.7 cells were quantified using ELISA kits (Bioswamp, Wuhan, China) in strict accordance with the manufacturer’s protocols.

#### Quantitative phagocytosis assay

To quantitatively assess phagocytic activity, the Vybrant ™ Phagocytosis Assay Kit (Molecular Probes, USA) was employed in accordance with the manufacturer’s protocol. RAW264.7 cells were seeded at a density of 6 × 10^3^ cells per well in a 96-well microtiter plate. After a 24-hour incubation period with the following treatments: IL-4 (20 ng/mL, ABclonal, USA), IL-4 + isoprenaline (ISO, 250 μM, Cayman, USA), IL-4 + ISO + ICI118,551 (10 μM, GLPBIO, USA), the cells were subsequently co-incubated with 100 μL of fluorescein-labeled Escherichia coli (K-12 strain) bioparticles for 4 h. Following this incubation, the E. coli bioparticle suspension was aspirated, and extracellular fluorescence was quenched by the addition of 100 μL of Trypan Blue for 1 min. The Trypan Blue was then removed, and fluorescence was measured using a Tecan Spark Multimode Microplate Reader (TECAN, Austria) with excitation and emission wavelengths set at 485 nm and 538 nm, respectively.

#### Open field experiment

SMART V3.0 Small Animal Behavioral Video Recording and Analysis System (Version 3.0, Panlab, Spanish) were used to record animal movement trajectories and conduct parameter analysis. Mice were quickly placed with their backs to the experimenter in the central area of the open field box before the experimenter left immediately. The behavioral video recording software recorded the mice’s activities in the open field box for 10 min. After all animal experiments were completed, the video data was export to an Excel spreadsheet for analysis.

#### Elevated plus maze analysis

The mice were gently placed in the central area of the instruments. Animal behavior analysis of SMART V3.0 software was used to track the trajectories movement of mice within the elevated plus maze instrument automatically.

#### Barnes maze test

##### Habituation

Half an hour before testing, the mice were placed in the testing room to acclimate to the environment. The day before training and testing, the mice were allowed to habituate and familiarize themselves with the test board for 5 min. During this time, the mice were guided into the target box multiple times.

##### Training

The mice were placed in the center of the test board and cover it with a plastic bucket for 5 sec. After removing the bucket, the mice were allowed to freely explore. Each test lasted 3 min or until the mouse entered the target box, which completed one training session. If the mouse did not find the target box within 3 min, we manually placed the mouse in the target box and allowed it to stay there for 15 sec. After each training session, the test board and target box must be wiped with 75% alcohol. Two training sessions were conducted daily, with an interval of one hour, for a total of 5 days. The latency time for the mouse to enter the target box was recorded.

##### Testing

On the 6th day of the experiment, remove the target box. Record the time the mouse spends within the vicinity of the target box’s original location within a 3-minute period (JLBehv - BNSM, Shanghai, China).

#### Three-chamber test

##### Sociability

The testing apparatus was divided into three chambers with one side containing a stranger mouse of the same species (Stranger 1) in a metal cage, and the other side having only an empty metal cage. The mouse was allowed to freely explore the three chambers. Before the experiment began, the mouse was in the behavioral testing room to adapt for 30 min and placed in the middle chamber to adapt for 5 min. The same-sex stranger mouse (Stranger 1) was randomly placed into the metal cage in the left chamber, while leaving the metal cage in the right chamber empty. The partitions separating the chambers was removed, allowing the test mouse to freely move among the three chambers for 5 min. Immediately begin filming and recording relevant parameters using EthoVision XT (Noldus, Netherlands). The sociability preference index was calculated from the following formula [(time exploring the stranger 1 - time exploring the empty cage) / (time exploring the stranger 1 + time exploring the empty cage)]

##### Social novelty

To assess interest in social novelty, another stranger mouse (Stranger 2) was introduced into the empty cage as a social novelty stimulus. The interaction time between the test mouse and Stranger1 and Stranger 2 was recorded. The social novelty preference index was [(time exploring the Stranger 2 - time exploring the Stranger 1) / (time exploring the Stranger 2 + time exploring the Stranger 1)].

##### Social memory

Twenty-four hours after completing the first and second phases, stranger 2 was replaced with another new stranger mouse (Stranger 3) and recorded for 5 minutes. the Interaction frequency and time between the test mouse and Stranger 1 and Stranger 3 were recorded. The social memory preference index was [(time exploring the Stranger 1 - time exploring the Stranger 3) / (time exploring the Stranger 1 + time exploring the Stranger 3)].

#### CODA non-invasive blood pressure monitoring

The mice were placed in a support stand for monitoring. The O-cuff was slid towards the base of the tail until resistance was met, and then the VPR cuff was slid up from the tail towards the larger diameter end in front. Blood pressure was measured according to the manufacturer’s instructions. Body temperature was recorded before blood pressure measurement.

#### Establishment of peripheral sympathetic nerve elimination model

C57BL/6J mice were injected with 1 × 10^6^/100 μL LLC-LUC cells subcutaneously. Seven days after sleep deprivation, the mice were divided into two groups: a control group receiving normal saline and a group undergoing peripheral sympathetic nerve block, administered with 6-hydroxydopamine (100 mg/kg, Cayman, USA) intraperitoneally every 2 days. Following 12 days of sleep deprivation, tumor-bearing mice were sacrificed and the subcutaneous tumors were dissected, photographed, weighed and analyzed statistically.

#### Tumor models and PD-1 treatment

After subcutaneous injection of LLC-LUC cells into C57BL/6J mice, the mice were allocated into two distinct groups: one group maintained a regular sleep schedule as negative control (NC), while the other group was subjected to sleep deprivation (SD). When the tumor volume exceeded 100 mm^3^, the mice received intraperitoneal administration of PD-1 inhibitor (10 mg/kg, Bio X Cell, USA) every three days. Tumor volume was measured every three days using calipers. The animals were euthanized after 12 days of PD-1 inhibitor treatment.

#### Stereotaxic surgery

Adult PNMT-Cre transgenic mice (25 - 30 g) were anesthetized with pentobarbital sodium (60 μg/g, i.p.), and mice were bilaterally stereotaxically injected with AAV-hSyn-DIO-hM4Di-EGFP into the RVLM. The depth of anesthesia was evaluated by the disappearance of corneal reflex and hindpaw reflex. All surgeries were performed under strict aseptic conditions. After anesthesia, the mice were placed prone on a stereotaxic apparatus (RWD, China), and their body temperature was maintained at 37°C with a heating pad. The dorsal surface of the RVLM was exposed by craniotomy. A glass micropipette containing the virus vectors was placed on a syringe pump (Harvard Apparatus, USA), and the virus vectors were injected bilaterally into the RVLM according to the positioning. AAV virus vectors were slowly injected into the RVLM (bregma: anteroposterior -6.50 mm to -6.65 mm; mediolateral ± 1.28 mm; dorsoventral -6.50 mm) under pressure. The pipette was left in place for at least 5 min before withdrawal. After injection, mice were injected with antibiotic ampicillin (125 mg/kg, i.p.) and analgesic ketorolac (4 mg/kg, i.p.). The surgical process and virus injection did not produce observable behavioral effects, and a 2-week recovery period was conducted before the sleep deprivation experiment. For the chemogenetics experiment, the mice intraperitoneally received clozapine N-oxide (CNO, 2.0 mg/kg, Tocris, USA) daily two weeks after hM4D virus injection. The virus titers were 1.3 × 10^12^ vg/mL.

#### Immunofluorescence assay

Under the anesthesia of pentylenetetrazol, mice were perfused transcardially with chilled saline, followed by 4% paraformaldehyde. Brains were post-fixed in 4% paraformaldehyde at 4°C for 24 - 48 h, then dehydrated in 30% sucrose solution for 48 h. Brain sections were made along the coronal plane at a thickness of 25 μm on a freezing microtome (Leica Microsystems, Germany). Then the sections were washed with PBS (3 × 5 min). The sections were blocked in 5% bovine serum albumin (BSA) in 0.25% Triton X-100 in PBS for 30 min at room temperature, followed by incubation with primary antibodies in 2% BSA-PBS overnight at 4°C. The following primary antibodies were used: guinea pig anti-cFos (Abcam, UK), rabbit anti-TH (Abcam). After sufficient washing in PBS, sections were incubated in proper fluorescently conjugated secondary antibodies for 2 h at room temperature over a shaker at low speed. Finally, sections were mounted on slides. Fluorescence images were taken with a confocal microscope (Zeiss, Germany) and processed with ZEN software (Zeiss). Cells were manually counted in confocal images.

#### Multiparameter flow cytometry

The spleen and tumor tissue were placed in PBS, cut into 2 - 4 mm small pieces, digested with 0.1% collagenase I, IV, and 0.002% DNaseI enzyme at 37°C in a water bath for 30 min, vigorously shaken every 5 min, filtered through a 40 μm Falcon cell strainer to remove undigested tumor tissues, and then added to DMEM complete culture medium to terminate the enzymatic reaction. Tissue suspension was centrifuged and the supernatant was discarded.

Red blood cells were lysed with ACK lysis buffer.at room temperature and incubated for 5 min. After adding an equal volume of PBS to terminate the digestion, the supernatant was centrifuged and the supernatant was discarded. PBS was used to suspend the cell precipitate, and CD16/32 antibody was added to avoid nonspecific antibody binding, incubated at 4°C for 5 - 10 min. The cell suspension was then divided into two parts for macrophage staining and T-cell staining. T-cell staining was performed with fluorophore-conjugated antibodies (FITC-conjugated anti-mouse CD3, APC-Cy7-conjugated anti-mouse CD4, PerCP-conjugated anti-mouse CD8, APC-conjugated anti-mouse CD25, PE-conjugated anti-mouse ADRB2, and PE-Cy7-conjugated anti-mouse NK1.1). For macrophage staining, the cell suspension was incubated with antibodies specific for APC-Cy7-conjugated anti-mouse CD45, FITC-conjugated anti-mouse CD11b, PE-Cy7-conjugated anti-mouse CD86, and PerCP-Cy5.5-conjugated anti-mouse F4/80. For CD206 staining (APC-conjugated anti-mouse CD206), Cytofix/Cytoperm kit (BD, USA) was used for fixation and permeabilization, All the samples were performed using CytoFLEX LX (Beckman Coulter, USA) and the data were analyzed with FlowJo software or CytExpert.

#### Cytokine antibody array

A mouse cytokine array (QAM-CYT-1-1; RayBiotech, Norcross, GA, USA) was utilized to assess the secretion levels of 22 cytokines in the serum of sleep-deprived tumor-bearing mice and NC tumor-bearing mice following the manufacturer’s instructions. Quantitative array analysis was performed using the ImageQuant LAS4000 Scanner (GE, Boston, MA, USA). Cytokines were screened using the following integrated conditions: Fold change ≥ 1.2 and fluorescence intensity values > 300.

#### Flow cytometry mass spectrometry

The key effector cells responsible for the protumoral immunity induced by sleep deprivation were identified through CyTOF experiments. CyTOF sample preparation: 3 × 10^6^ cells were added to the flow cytometry tube, and PBS (without calcium and magnesium) was washed twice (centrifuged at 500 g at room temperature for 5 min). Platinum dichloride was used to distinguish dead and live cells: 1 mL of a final concentration of 0.5 μM platinum dichloride was added, mixed well, and incubated at room temperature for 2 min.

2 mL of cell staining buffer was added and centrifuged at 4 °C at 800 g for 5 min. Subsequently, 500 μL of preservation solution (10% DMSO + 90% cell staining buffer) was added, and the sample was stored at -80°C for future use. Machine detection: follow the instructions for operation of the instrument to detect the distribution of each subtype of immune cells and the expression level of chemokines and cytokines.

#### Cell culture

LLC-LUC cells and RAW264.7 cells were cultured in complete DMEM supplemented with 10% fetal bovine serum, 1% penicillin‒streptomycin, and 1% L-glutamine. The culture supernatants of RAW264.7 cells treated with IL-4, IL-4 + ISO, IL-4 + ISO + ICI118,551 were added to DMEM culture medium containing 10% FBS at a 30% ratio for the culture of LLC-LUC cells, with 2 mL of the mixture per well. Suppression of STAT6 phosphorylation was performed by treatment with 100 nM AS1517499 (MCE, USA) for 24 h in IL-4 and ISO-stimulated RAW264.7 cells.

#### ADRB2 antagonist treatment

C57BL/6J mice were intravenously injected with 5 × 10^5^/100 μL LLC-LUC cells. After 7 days of sleep deprivation, the mice were divided into two groups: one receiving treatment with ADRB2 antagonist ICI118,551 intraperitoneally (10 mg/kg, GLPBIO, USA) and the other administered with saline, along with sleep deprivation. Flow cytometry of tumor tissue was performed after 21 days.

#### RNA interference

RAW264.7 cells were seeded at a density of 2 × 10^5^ cells per well in a 24-well plate and transfected with siRNA (20 nM) using Hieff Trans® *in vitro* siRNA/miRNA Transfection Reagent (Yeasen, Shanghai, China) according to the manufacturer’s instructions. The sequences of the siRNAs are provided in Table S2.

#### Quantitative reverse transcription PCR (qRT-PCR)

Briefly, total RNA was isolated using the Eastep TM Total RNA Kit (Promega, Beijing, China). Subsequently, cDNA synthesis was performed with HiScript III Reverse Transcriptase (Yeasen) and amplified using a Light Cycler 480 II Real-Time PCR System (Roche, Basel, Switzerland) in combination with Hieff UNICON® advanced qPCR SYBR Master Mix (Yeasen). GAPDH served as internal controls following the classical 2^-ΔΔCt^ method. The primers were designed and synthesized in Sangon Biotech (Shanghai, China). The primer sequences are listed in online Table S2.

#### Western blotting

RAW264.7 cells were washed with PBS and then lysed in a lysis solution containing radioimmunoprecipitation assay buffer (RIPA) and 1% PMSF. The cell lysates were subsequently boiled at 100°C for 5 min, separated by sodium dodecyl sulfate-polyacrylamide gel electrophoresis (SDS-PAGE), and transferred onto polyvinylidene fluoride (PVDF) membranes. After blocking the membranes with 5% BSA for 1 h, they were incubated with primary antibodies against KLF4 (Proteintech, Wuhan, China), mouse monoclonal antibody against JAK1 (Proteintech), rabbit polyclonal antibody against p-JAK1 (ImmunoWay, USA), mouse monoclonal antibody against STAT6 (Proteintech) and rabbit polyclonal antibody against p-STAT6 (ImmunoWay) for 2 h, followed by three washes with Tris-buffered saline containing Tween-20 (TBST), and then incubated with appropriate secondary antibodies. Visualization of the nitrocellulose membrane was achieved using enhanced chemiluminescence plus reagents and an imaging analysis system.

#### Immunohistochemistry (IHC)

The paraffin sections of mice tumor tissues were subjected to baking, dewaxing, dehydration, elimination of endogenous peroxidase activity, tissue pressure-resistant antigen repair, 5% BSA blocking, dropping of rabbit polyclonal antibody against KLF4 (Proteintech), rabbit polyclonal antibody against CD31 (Proteintech), rabbit polyclonal antibody against p-JAK1 (ImmunoWay), rabbit polyclonal antibody against p-STAT6 (ImmunoWay), PBS washing at 4°C overnight, incubation with secondary antibody at 37°C for 35 min, PBS washing, and staining. Next, the sections were then dehydrated and mounted. Last, the images were observed and scanned under a microscope. The tissue microarray chip (ZL-LUC1601) used to detect the expression of ADRB2 and KLF4 in NSCLC were purchased from Shanghai Weiao Biotechnology Co., Ltd.

#### Questionnaire analysis

131 NSCLC patients who received treatment in Department of Tumor Immunotherapy of Hebei Medical University were enrolled in this study to determine the effect of sleep deprivation on the NE level in serum. Pittsburgh Sleep Quality Index scale and sleep quality questionnaire were conducted to analyze the sleep status of the NSCLC patients. Sleep deprivation was defined as less than 6 h of sleep within 24 h, [25] and the NSCLC patients were divided into the sleep deprivation group and the NC group according to this definition. The concentration of NE in the serum of NSCLC patients was measured using ELISA.

### Quantification and statistical analysis

Statistical analysis was conducted and statistical graphics were drawn using GraphPad Prism v8.0.2 software. All measurement data are presented as the mean ± SD or mean ± SEM. Student’s t-test was used to analyze the data with equal variance, and the Mann-Whitney U test was used for those data without equal variance. *P* < 0.05 were considered statistically significant, and all *P* values were two-tailed.
