# Supplementary material for: Sleep deprivation-induced sympathetic activation promotes pro-tumoral macrophage phenotype via the ADRB2/KLF4 pathway to facilitate NSCLC metastasis
Source: iScience. 2025 Mar 30;28(5):112321. doi: 10.1016/j.isci.2025.112321 (PMC12018092; doi:10.1016/j.isci.2025.112321)
Supplement: Document S1. Figures S1–S6 and Tables S1 and S2 [file mmc1.pdf]

iScience, Volume *28*

Supplemental information

Sleep deprivation-induced sympathetic activation promotes pro-tumoral macrophage phenotype via the ADRB2/KLF4 pathway to facilitate NSCLC metastasis

Shuxian Yin, Jiali Wang, Yunlong Jia, Xiaoyi Wang, Yan Zhao, Tianxu Liu, Wei Lv, Yuqing Duan, Song Zhao, Sheng Wang, and Lihua Liu

Supplemental information

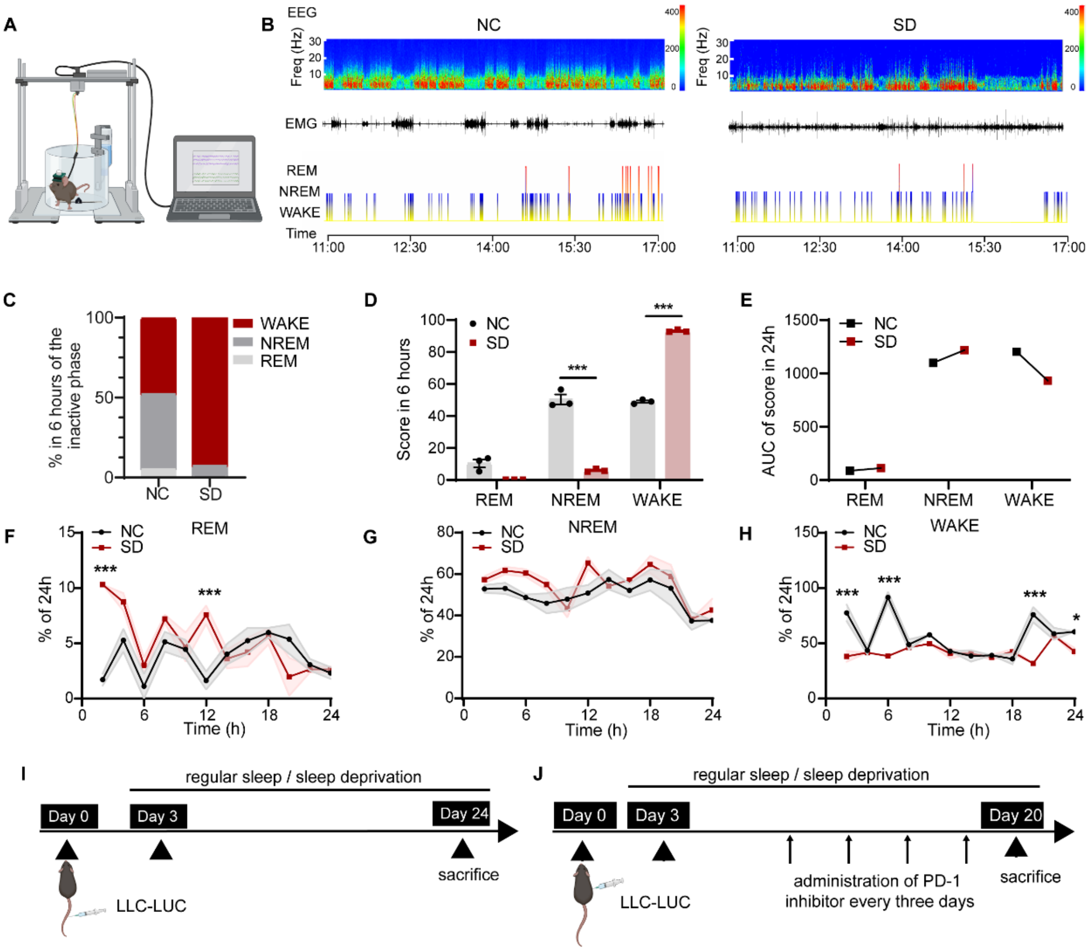

Supplementary Figure 1. Impact of sleep deprivation on sleep patterns, related to Figure 1. **(A)** Schematic diagram of EEG/EMG recordings during sleep deprivation in tumor-bearing mice. **(B and C)** Sleep monitoring during normal sleep and sleep deprivation periods in mice. **(D)** Percentage of REM, NREM, and awake time in the NC group and sleep deprivation group from 11:00 A.M. to 5:00 P.M. (n = 3). **(E)** Comparison of REM, NREM, and awake time during sleep recovery period in the NC group and sleep deprivation group. Proportions of REM **(F)**, NREM **(G)** and wake **(H)** within 24 hours after sleep intervention. **(I)** Schematic illustration of NSCLC metastatic model with sleep deprivation. **(J)** Schematic diagram of PD-1 inhibitor during sleep deprivation. All the data are shown as the mean ± SEM. Statistical significance was calculated using a two-tailed unpaired Student's t test or Mann-Whitney U test. **P* < 0.05, ****P* < 0.001.

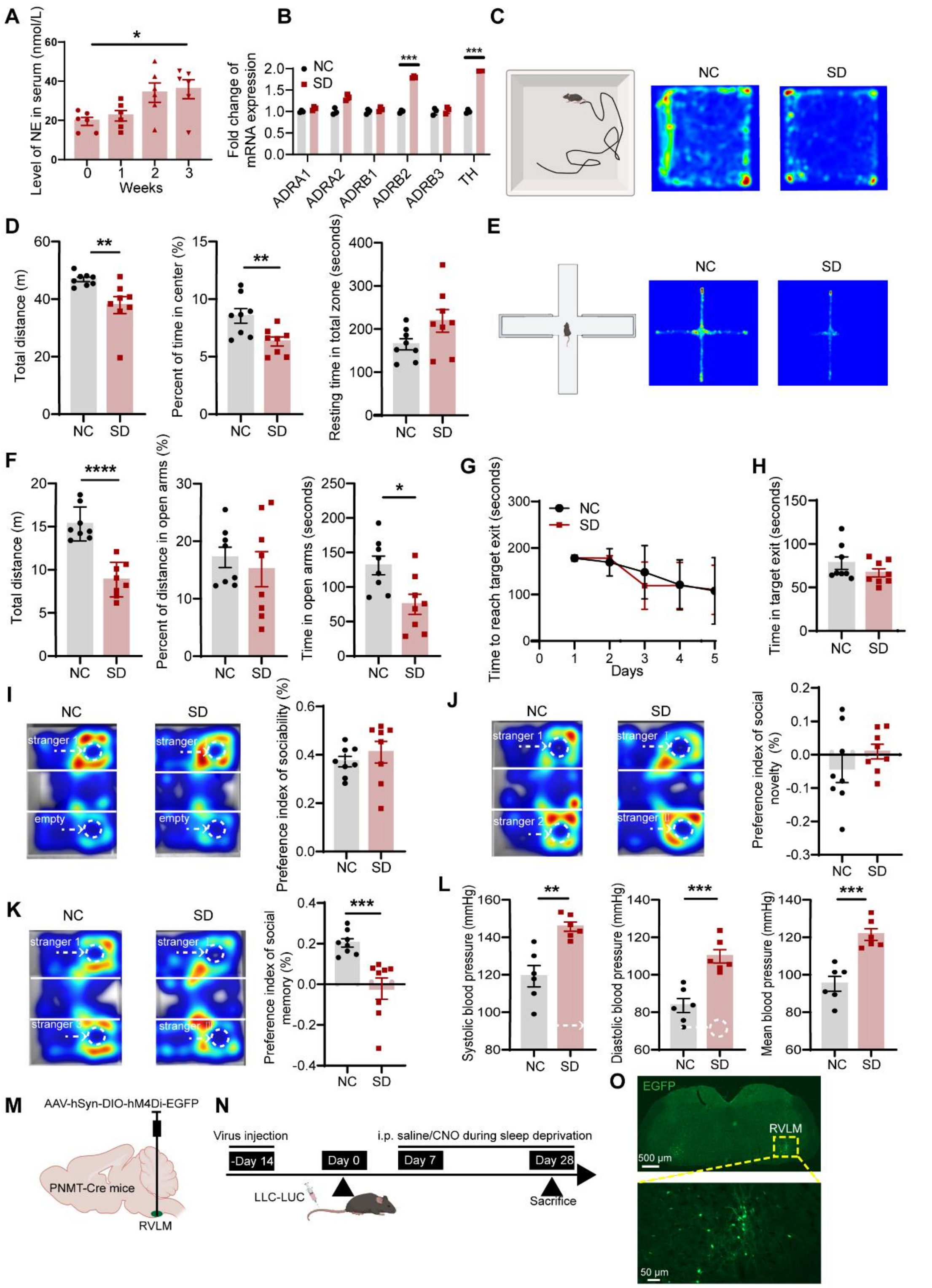

Supplementary Figure 2. Effects of sleep deprivation on sympathetic system and behavioral responses, related to Figure 2. **(A)** Expression of NE during sleep deprivation (n = 6). **(B)** qRT-PCR analysis of adrenergic receptor and tyrosine hydroxylase in tumor tissue (n = 3). Representative images of open field test **(C)** and histogram analysis of behavior data for open

field test **(D)**. Representative images of elevated plus maze test **(E)** and histogram analysis **(F)** of behavior data for elevated plus maze test between the two groups. (n = 8). **(G)** Duration in target exit area in Barnes maze testing. **(H)** Graphs illustrating the time taken to reach the designated exit target during the exercise period. Evaluation of social ability **(I)**, social novelty **(J)** and social memory **(K)** in three-chamber social test. **(L)** Histograms.depicting systolic blood pressure, diastolic blood pressure and mean blood pressure under the indicated conditions (n = 6). **(M)** Schematic diagram of viral microinjection into the RVLM of PNMT-Cre mice. **(N)** Experimental design: PNMT-Cre mice transfected with hM4Di-EGFP were inoculated and subjected to sleep deprivation, during which they received either saline or CNO. **(O)** Fluorescent validation of hSyn-DIO-hM4Di-EGFP virus transfection of PNMTRVLM neurons. Statistical analyses used the Student's t test or Mann-Whitney U test. **P* < 0.05, ***P* < 0.01, ****P* < 0.001.

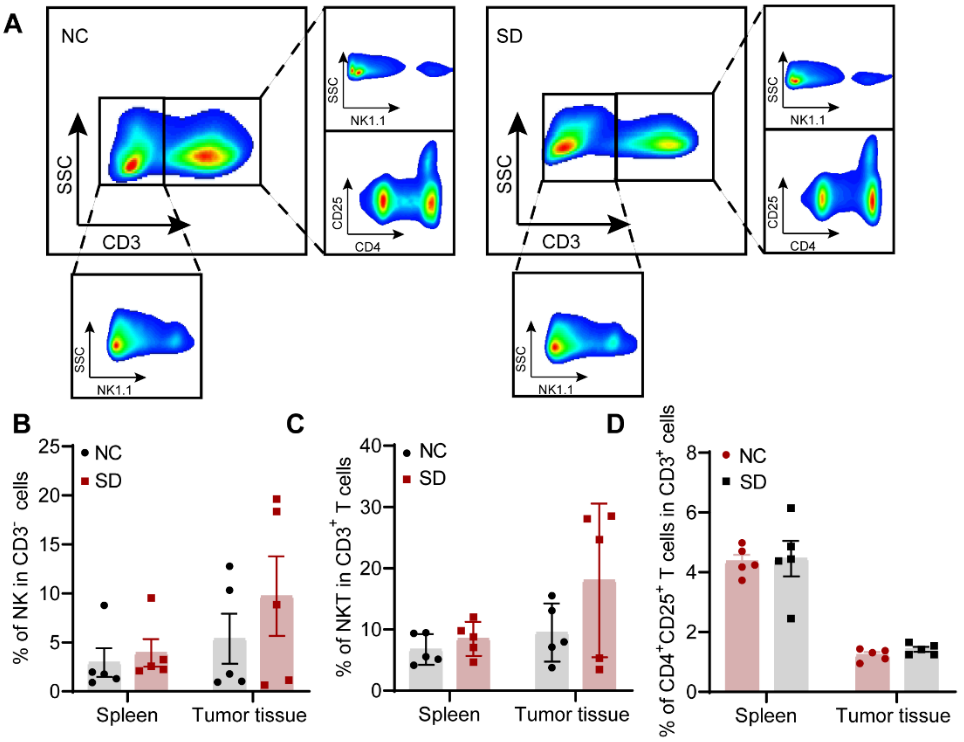

Supplementary Figure 3. Flow cytometric analysis of NK, NKT, and Treg cell populations: gating strategies and expression profiles, related to Figure 3. **(A)** Gating strategies for flow cytometric analysis of NK (CD3-NK1.1+), NKT (CD3+NK1.1+) and Treg (CD3+CD4+CD25+) cells. **(B - D)** Histograms depicting the expression of NK, NKT and Treg cells. Statistical significance was determined through the Student's t-test or Mann-Whitney U test.

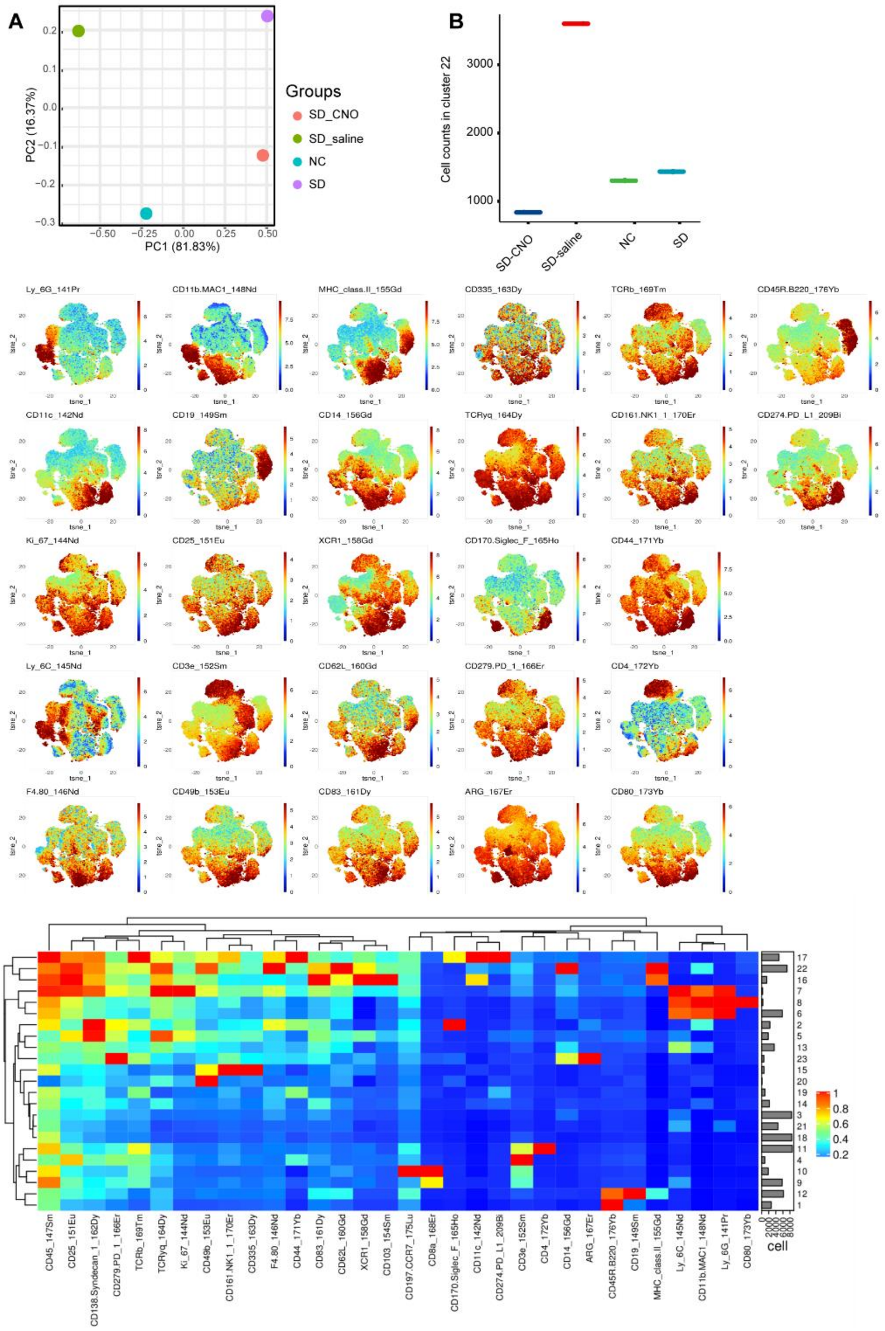

Supplementary **Figure 4.** Detailed information of flow cytometry mass spectrometry, related to Figure 4. **(A)** The principal component analysis of differences in samples from indicated groups. **(B)** Comparison of macrophage counts in different groups. **(C)** t-SNE heatmap of marker signal values for samples. **(D)** Heatmap of marker distribution in each cluster.

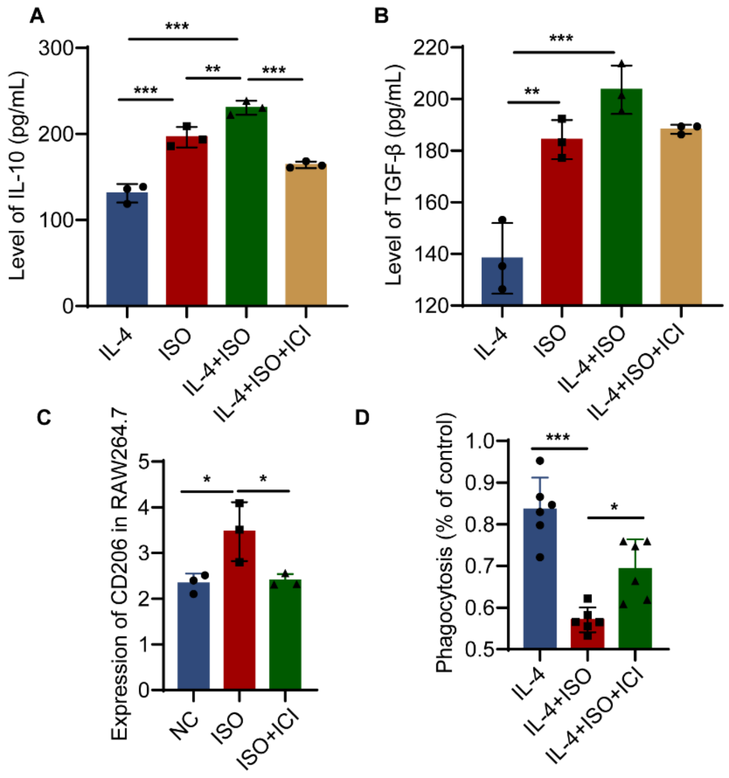

Supplementary Figure 5. Effect of ADRB2 on macrophages, related to Figure 5. **(A**, **B)** Expression of IL-10 and TGF-β in conditioned medium. **(C)** Assessing CD206 expression levels through flow cytometric analysis. **(D)** Phagocytic function of macrophages under different treatment conditions. One-way ANOVA was performed and p values were calculated using Tukey's multiple comparison test. $^{*}P < 0.05$, $^{**}P < 0.01$, $^{***}P < 0.001$.

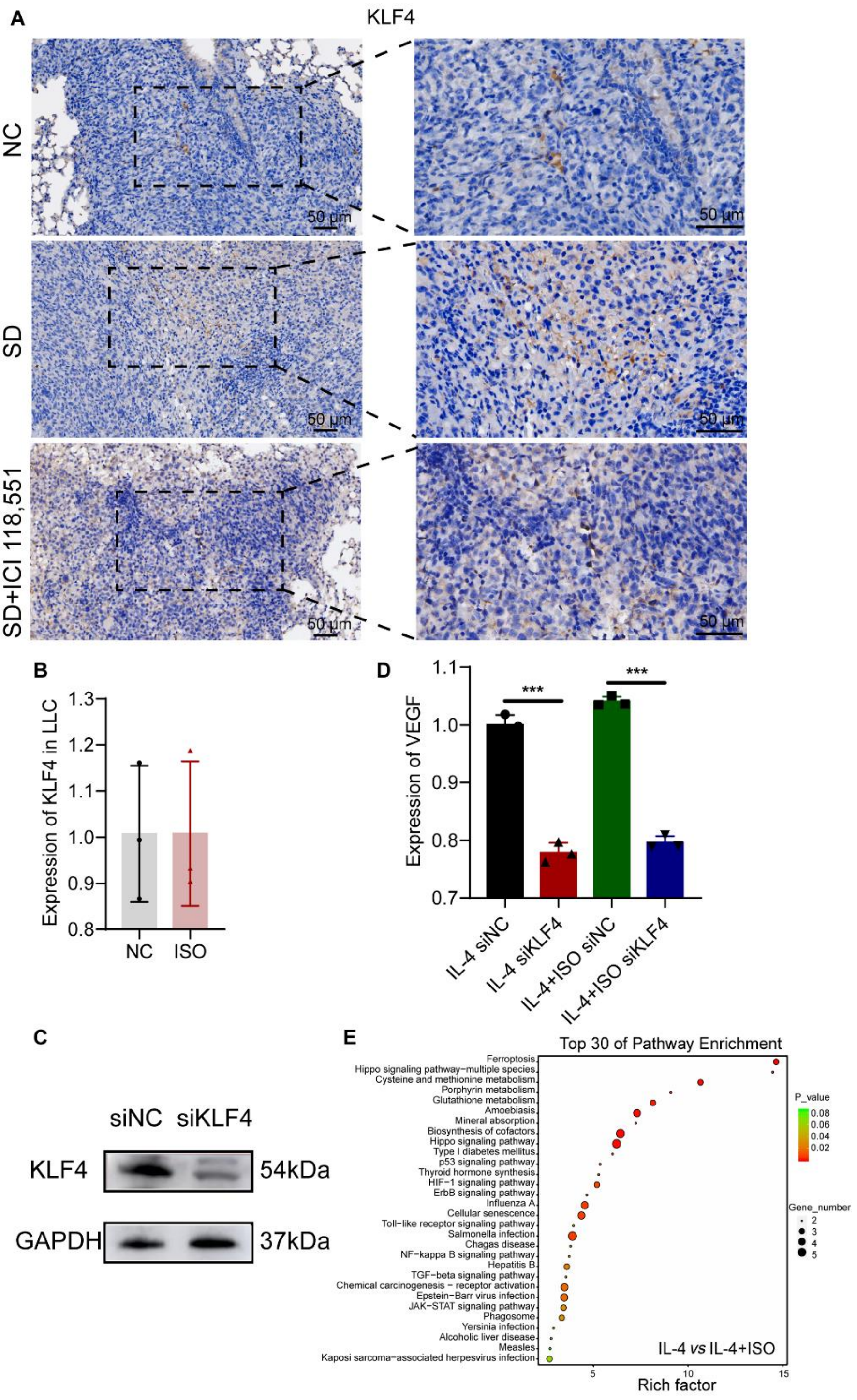

Supplementary Figure 6. Role of KLF4 in tumor microenvironment and macrophage polarization, related to Figure 6. **(A)** The expression of KLF4 in tumor tissues. **(B)** qRT-PCR analysis of KLF4 in LLC with or without ISO intervention. **(C)** Western blotting verification of KLF4 in RAW264.7 transfected with siRNA. **(D)** qRT-PCR analysis of VEGF in RAW264.7 treated with or without ISO intervention. **(E)** Top 30 pathway enrichment in differentially expressed genes in IL-4-treated macrophages with ADRB2 agonist or not. Statistical significance was determined through the Student's t-test or Mann-Whitney U test. $^{***}P < 0.001$.

Table S1 Clinicopathological parameters of NSCLC patients and correlation with ADRB2 expression (n=80)

Clinicopathological parameters	N of cases	ADRB2 expression		P value
		High	Low	
Gerder				0.1053
Male	30	19	11	
Female	50	21	29	
Age				>0.999
≤60	43	22	21	
>60	37	18	19	
Differentiation grade				0.8213
Well	22	10	12	
Moderate+Poor	58	30	28	
AJCC stage				0.01
I+II	46	17	29	
III+IV	34	23	11	
Lymph node metastasis				0.6367
Negative	53	28	25	
Positive	27	12	15	

Table S2 Primer sequences and siRNA sequences

Gene	Forward 5'-3'	Reverse 3'-5'
GAPDH	TGGCCTTCCGTGTTCCTAC	GAGTTGCTGTTGAAGTCGCA
ADRA1A	CTGCCATTCTTCCTCGTGAT	GCTTGGAAGACTGCCTTCTG
ADRA2A	TGCTGGTTGTTGTGGTTGTT	GGGGGTGTGGAGGAGATAAT
ADRB1	TCGCTACCAGAGTTTGCTGA	GGCACGTAGAAGGAGACGAC
ADRB2	TGGTTGGGCTACGTCAACTC	CCAGCTGACAAGTGTTTGGC
ADRB3	TGAAACAGCAGACAGGGACA	TCAGCTTCCCTCCATCTCAC
TH	GTCTCAGAGCAGGATACCAAGC	CTCTCCTCGAATACCACAGCC
KLF4	CTATGCAGGCTGTGGCAAAACC	TTGCGGTAGTGCCTGGTCAGTT
KLF9	GCCGCCTACATGGACTTCG	GGTCACCGTGTTCCTTGGT
HECW2	TTTTTGTGCGGCGTCGAAATC	CATGTTCTCGGGCATTGAGTT
EFNB1	TGTGGCTATGGTCGTGCTG	TCTTCGGGTAGATCACCAAGC
TBC1D8	TTGCTCCATTTCGGATTCTGC	CCAGTGCCGGTTGATTTCCT
IL-13	CCTGGCTCTTGCTTGCCTT	GGTCTTGTGTGATGTTGCTCA
IL-10	GGAGAACCTGAAGACCCT	GGCTTTGTAGATGCCTTTC
TGF-β	GGCCAGATCCTGTCCAAGC	GTGGGTTTCCACCATTAGCAC
VEGF	CCAGCTGACAAGTGTTTGG	GGCGATTTAGCAGCAGATATAAGAA
Si-KLF4-1	GCAGCTTGCAGCAGTAACA	
Si-KLF4-2	CGGAGTTGGACCCAGTATA	
Si-KLF4-3	CTATGCAGGCTGTGGCAAA	
